# Optimal Molecular Design: Generative Active Learning Combining REINVENT with Precise Binding Free Energy Ranking Simulations

**DOI:** 10.1021/acs.jctc.4c00576

**Published:** 2024-09-03

**Authors:** Hannes H. Loeffler, Shunzhou Wan, Marco Klähn, Agastya P. Bhati, Peter V. Coveney

**Affiliations:** † Molecular AI, Discovery Sciences, R&D, AstraZeneca, Mölndal 431 83, Sweden; ‡ Centre for Computational Science, Department of Chemistry, University College London, London WC1H 0AJ, U.K.; § Advanced Research Computing Centre, University College London, London WC1H 0AJ, U.K.; ∥ Institute for Informatics, Faculty of Science, University of Amsterdam, Amsterdam 1098XH, The Netherlands

## Abstract

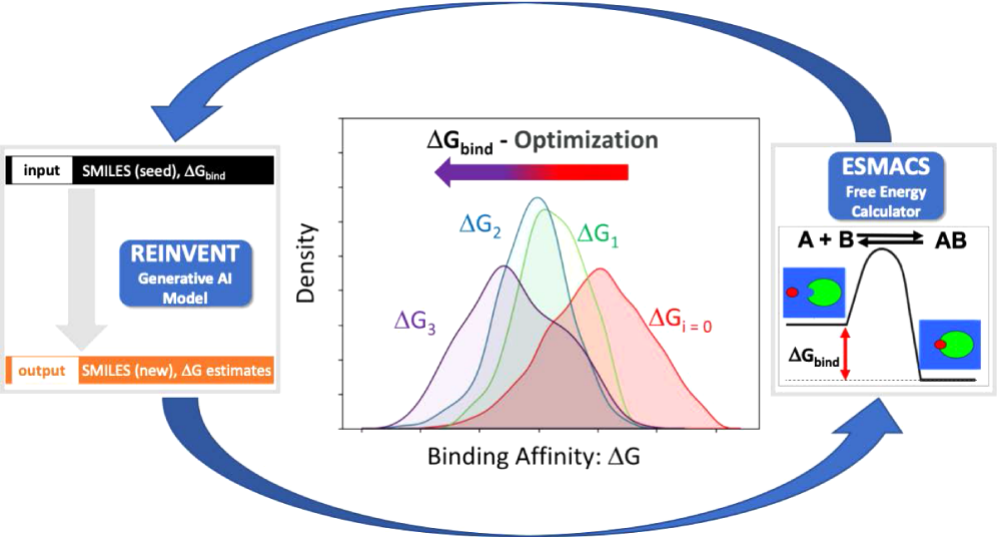

Active learning (AL) is a specific instance of sequential experimental design and uses machine learning to intelligently choose the next data point or batch of molecular structures to be evaluated. In this sense, it closely mimics the iterative design-make-test-analysis cycle of laboratory experiments to find optimized compounds for a given design task. Here, we describe an AL protocol which combines generative molecular AI, using REINVENT, and physics-based absolute binding free energy molecular dynamics simulation, using ESMACS, to discover new ligands for two different target proteins, 3CL^pro^ and TNKS2. We have deployed our generative active learning (GAL) protocol on Frontier, the world’s only exa-scale machine. We show that the protocol can find higher-scoring molecules compared to the baseline, a surrogate ML docking model for 3CL^pro^ and compounds with experimentally determined binding affinities for TNKS2. The ligands found are also chemically diverse and occupy a different chemical space than the baseline. We vary the batch sizes that are put forward for free energy assessment in each GAL cycle to assess the impact on their efficiency on the GAL protocol and recommend their optimal values in different scenarios. Overall, we demonstrate a powerful capability of the combination of physics-based and AI methods which yields effective chemical space sampling at an unprecedented scale and is of immediate and direct relevance to modern, data-driven drug discovery.

## 1 Introduction

Designing optimized molecules for specific purposes is fundamental to chemistry and is central to the discovery of new medicines and new materials. In practice, this is an iterative, slow, and expensive process^1,2^ commonly achieved through a DMTA (design-make-test-analysis) cycle where newly created compounds need to be tested for their properties and inform the design for the next iteration^3,4^·Thus, there is a natural desire to turn to computation to find better compounds more quickly and cheaply.

Active learning (AL) is a commonly applied machine learning (ML) method that queries an information source, which could be a human expert (human-in-the-loop) or accurate computational predictor, to label large amounts of data interactively. An AL algorithm seeks to find an optimal way to label only a small subset of the data out of a vast amount of unlabeled data and thus accelerate the learning process. The subset is selected actively, i.e. intelligently, to minimize the number of iterations and only forward samples to the expert which the algorithm predicts will increase knowledge or is uncertain about. A passive algorithm to construct a prediction model would choose the subset at random and often not in an iterative manner. AL is thus classed as a semisupervised learning strategy as the expert can only explicitly label a small amount of the data set. These labels inform a predictive model (the surrogate) which can eventually be used to label the whole data set as needed.

The relationship of AL to Bayesian optimization^5^ (BO) has been noted, in particular to pool-based AL.^6^ AL algorithms define an efficient way to label data and so improve the predictive model while BO seeks to find the global optimum of an unknown (“black box”) function. In pool-based AL, the distribution of the data is unknown and consequently only a discrete subset of examples can be queried rather than the whole data set being available upfront. In contrast, in population-based AL the distribution is known, and the goal is to find the optimal density of the distribution.^7^ Here, we will discuss an algorithm that seeks to optimize for ligand-protein binding affinity while still maintaining a high level of exploration (that is, of diversity to promote discovery of a wide range of new molecules).

AL for molecule design often makes use of large libraries or vendor catalogs as the pool,^8−10^ when the size of the data set is known a priori. The size of these libraries can easily range into the millions and, indeed, even billions of compounds.^11^ In AL a, typically, computationally expensive method like docking or MD simulation is used to assign new labels e.g., a free energy of binding (also known as the binding affinity) or a docking score as proxy for the affinity. AL approaches that efficiently optimize compounds for binding affinity with RBFE (relative binding free energy) methods (commercial software vendors frequently refer to this as a “FEP” method) have only appeared recently in the literature^12−20^ but have also been used to optimize an RBFE protocol itself.^17^ Combining Generative AI with active learning (GAL) has only started very recently^21^ including a proof-of-concept study with peptides^22^ and an application with ABFE^23^ (absolute binding free energy). The latter focuses on multifidelity surrogate modeling where training data is taken from docking, experimental results from BindingDB,^24^ and a double-decoupling ABFE method.^25^

AL relies on an oracle (a term which refers to either a human expert or a more expensive computational method) for labeling and is the ground-truth in this scheme. A surrogate model is created with the aim to reproduce the predictions of the oracle but at a much lower computational cost. Typical algorithms used here are classical ML methods used in QSAR modeling such as random forest or state vector machines, but they can also be more sophisticated artificial neural networks or a Gaussian process, the latter especially being often the preferred choice with Bayesian optimization. The surrogate model can then be used to compute a much larger subset of the library or even its entirety to create the labels and so eventually replaces the expensive oracle. An acquisition function (often called infill sampling criteria in BO) would then be applied to select a new, small subset for evaluation with the oracle. Informativeness, representativeness and diversity have been proposed previously as desired criteria for the acquisition function.^26^ The quality of the surrogate model and the acquisition strategy are crucial to the design of the AL algorithm as the final goal, as in this study, is to find optimized molecules while using as little resources and/or time as possible. Alternatively, the final goal may be the construction of an optimal surrogate model to replace the oracle in making further predictions—for instance, the surrogate model could be used as the starting model for a related target.

In this work we replace the fixed-size library with a generative model to create molecules on-the-fly, drawing from a distributional description^27^ of chemical space.^28^ A generative model can produce a substantial subset of a chemical space^29^ whereas vendor libraries are naturally limited to the molecules contained in the library which is defined and restrained in terms of their synthesis protocols.^11^ To this end, we apply REINVENT^28^ which uses reinforcement learning (RL) to generate optimal molecules subject to external “information” i.e., scoring functions which evaluate each compound for its fitness. The RL algorithm drives a “prior” model of general chemical knowledge toward a specialized model representing the chemical space of the task (objective) at hand. The scoring function can be an agglomeration (weighted arithmetical or geometrical mean) of scoring components. The surrogate model is here the most crucial scoring component as it informs the RL algorithm about the label which in this case is the binding free energy of the compound to the target as estimated by ESMACS (enhanced sampling of molecular dynamics with approximation of continuum solvent).^46,47^ We note here that it is conventional in the community to call MMPBSA results “absolute binding free energies” but their absolute values are nonphysical and should rather be thought of as scores. Other, secondary, scoring components are also used; these are described in the Methods Section.

Our work demonstrates the power of combining AL with physics-based methods to effectively sample the vast chemical space. The major advantages of our approach over previous, similar approaches are (a) the novelty of generating high-quality small molecules with generative AI, (b) the innovation of combining this with a physics-based model in an AL workflow and thus the direct importance to in silico drug discovery, (c) the reliability of our physics-based oracle which is based on ensemble simulations that minimizes false positives/negatives, (d) the scale of operation using a batch size as large as 1000 to provide a more comprehensive picture, (e) deployment on very large scale HPC platforms such as Frontier, and (f) short wall clock time requirements.

## 2 Methods

Here we describe the detailed protocol for RL with REINVENT, the simulation protocol for ESMACS, the GAL workflow and provide some details on the protein targets used in this work.

### 2.1 Sampling Chemical Space with REINVENT

REINVENT’s main run mode is reinforcement learning (RL).^28^ With this approach a model (the agent) is iteratively biased toward a target profile which is the aggregation of scoring components describing the desirability of a molecule. Here we use the weighted geometric mean to aggregate the components into one total score for each generated molecule. The main scoring component is a QSAR-like response model (scoring weight of 0.6) created using ChemProp^30−33^ 1.5.2. ChemProp uses a directed message-passing neural network (D-MPNN) to produce a property prediction model. This model serves here as a surrogate model, and it is fixed in its algorithm and hyper-parameters. We thus assume that the model’s predictive power will remain mostly constant. It is updated with a new batch of molecules with predicted binding affinities from ESMACS simulations in each GAL iteration. The initial model for 3CL^pro^ is based on about 10 000 structures which have been found with a surrogate docking model.^34,35^ This ChemProp model was created after hyper-parameter optimization using cross-validation^36^ with 5 folds and 5 ensembles (5 models with different initial weight setup) each, the maximum number of iterations being set to 30. The initial data set was split in the ratio 0.8:0.1:0.1 for training, test, and validation, respectively. RDKit 2D normalization without feature scaling^32^ was used for this step as well as for all model updates. In each GAL step we update the model as described in Section 2.3. See ref (28) for details of the REINVENT protocol.

To bootstrap a model for TNKS2 the 27 known compounds with experimental affinity measurements from the benchmark set^37^ were handled with QSARtuna^38^ which allows automatic model selection from a series of classical machine learning algorithms. The best QSARtuna model was a random forest model which was subsequently used to generate 10 000 structures with REINVENT for evaluation with ESMACS (described in Section 2.2). The resulting binding free energies were used to train another ChemProp model. The initial model was created in the same way as the one for 3CL^pro^ and updated as described in 2.3.

The three other scoring components were QED,^39^ stereochemistry, both with weight 0.2, and structural alerts which act as a filter meaning that compounds which match an undesired structural pattern receive a score of zero or one otherwise. QED assigns a drug-likeness score to each molecule. This is needed to produce reasonable drug-like molecules and stay within the applicability domain of the ChemProp model as generative models very quickly optimize toward the weaknesses of a response model. For example, we found that if molecule generation were otherwise unrestrained, REINVENT would start creating molecules with very long alkyl chains because the ChemProp model scores these highly. This is, however, clearly undesirable. All weights have been adjusted manually in preliminary test runs to strike a balance between generation of unreasonable compounds and the main goal of generating molecules with better binding free energy. As the REINVENT prior does not support stereochemistry, molecules with stereocenters are scored with zero to encourage the agent to generate compounds without stereocenters. Structural alerts are a small set of SMARTS pattern to filter and suppress unwanted substructures (functional groups, ring sizes); see input configuration in the Supporting Information for details.

RL was run in two stages.^28^ The first stage uses QED, stereocenter and structural alert scoring components to create a sensible drug-like agent to reduce the need to query the surrogate model with less useful compounds. This only needs to be done once as preparation for the GAL protocol. In the second stage these three scoring components together with the ChemProp model were applied. Only this second stage was run in each GAL step. For molecule generation we use the classical Reinvent prior^40,41^ which is a de novo model that creates SMILES sequences from scratch. The number of new compounds generated in each GAL step is termed “batch” and its size was set to 100. As the learning strategy, we use the DAP^42^ with a sigma of 128 and a learning rate of 0.0001. A diversity scaffold filter was used to encourage exploration of a wide range of unique scaffolds. The scaffolds were generated using the Bemis-Murcko algorithm^43^ as implemented in RDKit.^44^ The diversity filter used a memory size of 10 scaffolds and enforced a minimum score of 0.7, meaning that molecules having that particular scaffold receive a zero score in the following steps, provided the total score exceeds 0.7. Identical SMILES were downscored to zero starting with the second occurrence in the RL run. An inception memory was used as replay memory^45^ with a memory size of 50 and a sample size of 10. In case of 3CL^pro^ we seeded the inception memory with the highest-scoring molecules of the current GAL iteration; in the case of TNKS2 the memory was seeded with the 27 experimental verified structures. The input configurations are shown in the Supporting Information.

### 2.2 Binding Affinity Prediction with ESMACS

We use the ESMACS protocol^46,47^ for the binding free energy calculations. ESMACS is based on MMPBSA (molecular mechanics Poisson–Boltzmann surface area)^48^ calculations but uses ensembles of replicas to obtain reproducible binding affinity estimates with robust uncertainty estimates. It is an end point free energy calculation, in which the binding free energy is calculated as the free energy changes of bound (compound-protein complex) and unbound (separated protein and compounds) states. Here conformations of the complex, protein and compound are all extracted from simulation of the complex, a commonly used protocol for the end-point free energy methods. Such a protocol is well suited for a rational drug screening project, in which the correct ranking of binding affinities is more important than the calculation of accurate binding affinities for the selection of compounds for further investigation.

#### 2.2.1 Model Preparation

The compounds generated from REINVENT were first processed using FixpKa^49^ 2.1.3.0 to obtain the correct protonation state at pH 7.4. Subsequently, up to 200 conformers per compound were generated using OMEGA^49,50^ 4.1.2.0. The prepared structural library of the compounds was then docked to the protein (PDB IDs: 6W63 for 3CL^pro^ and 4UI5 for TNKS2) using FRED,^49,51,52^ 4.1.1.0. The docking poses with the best Chemgauss4 docking scores were used for the ensuing ESMACS simulations.

Preparation and setup of the ESMACS simulations were implemented using BAC^53^ (binding affinity calculator). The AmberTools23 package^54^ was used for the setup of the systems and the calculations of binding free energies. Parameterizations of the compounds were produced using the general Amber force field 2 (GAFF2). The Amber ff14SB force field was used for the protein, and TIP3P for water molecules. Partial charges of the compounds were generated using the AM1-BCC method. The protonation states of the protein residues were assigned using the “reduce” module of AmberTools.^54^ All water molecules in the pdb files were retained. All ligand-protein complexes were solvated in orthorhombic water boxes with a minimum distance from the protein of 10 Å. Counterions were added to electrostatically neutralize the systems.

#### 2.2.2 Molecular Simulation

The standard ESMACS protocol^47^ uses an ensemble of 25 replicas for each compound-protein complex to get precise predictions. For a drug screening project, a vast number of compounds needs to be evaluated. A coarse-grained protocol may be used on grounds of speed, node hours required and low throughput due to the many ligands.^56^ Here we used an ensemble of 10 replicas, with a smaller box size and a buffer distance of 10 Å instead of 14 Å compared to that in the standard ESMACS protocol. Such a coarse-grained protocol inevitably decreases the precision of the predictions but is well-suited for high-throughput virtual screening.^56^ NAMD3^57^ was used as the MD engine for all of the equilibration and production runs. For each individual simulation, energy minimizations were first performed with heavy protein atoms restrained at their initial positions. The initial velocities were then generated independently from a Maxwell–Boltzmann distribution at 50 K, and the systems were heated up to and maintained at 300 K for 60 ps. Finally, 4 ns production simulations were run for each replica. For this study, we ran ∼17 440 and ∼22 000 ESMACS calculations overall for 3CL^pro^ and TNKS2, respectively, aggregating to a total simulation time of ∼2 ms. However, we would like to point out here that all these calculations were performed on the world’s first exascale machine, Frontier, allowing us to run them all concurrently for each iteration. Given that the performance of MD simulations for our systems was 150 ns/day using a single GPU (AMD Instinct MI250X), we were able to get results for the entire batch of compounds in 50 min of wall clock time for each iteration. Further, we note that the present study forms part of a much bigger workflow involving multiple additional steps (to be published elsewhere).

### 2.3 Generative Active Learning

**Figure 1:**
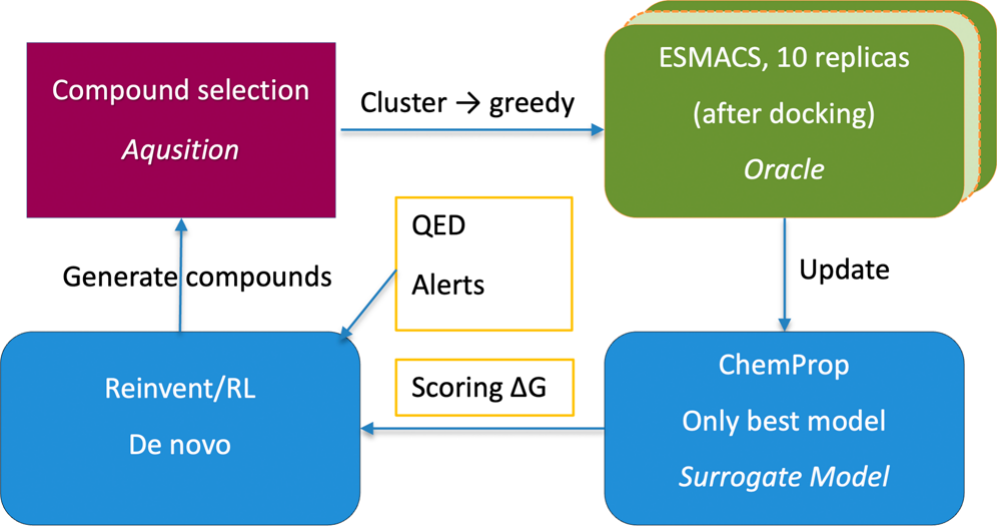
Schematic of overall workflow. Starting from the top right, ESMACS assesses the binding affinity of a set of compounds which are then used together with their Δ*G*_ESMACS_ to update a predictive ChemProp model (bottom right). REINVENT using the classical Reinvent prior recruits this model together with other scoring components (QED, Alerts, Stereo) to generate tens of thousands of new, potentially high-scoring molecules (bottom left). The acquisition step (top left) chooses a subset of the generated compounds by clustering and selecting the predicted highest-scoring molecules by Δ*G*_ESMACS_ (greedy selection) to feed into the next GAL iteration.

The main components of the GAL loop (see Figure 1) are ESMACS as the oracle, ChemProp as the surrogate model while REINVENT generates molecules on-the-fly and so replaces the more common fixed-size library,^12,14,58^ After evaluation of the current batch with ESMACS the surrogate model is updated. In principle, it is possible to freeze a selected set of layers in a ChemProp model to avoid “catastrophic forgetting”^59^ (also catastrophic interference). However, we found that training a new model from scratch from the accumulating set of molecules from each GAL cycle yielded somewhat better results than selectively freezing neural network layers. This is of course more expensive to train but it may also counter covariate shift to some degree. We used 5-fold cross validation and 5 ensembles in each fold to create the surrogate model. Model update was started from the model of the previous step such that no more than 6 epochs (model optimization iterations) were needed. But for performance reasons we only used the best model out of these 25 models as determined by the smallest test RMSE which, overall, stayed basically constant in all models built. RL was then run with the ChemProp model as free energy predictor together with the other scoring components (see above) in 300–500 iterations. REINVENT forms the inner optimization loop where RL is used for molecule optimization while the outer loop is the AL algorithm itself (see Figure 1).

The main parameters for GAL were the choice of the batch size and the selection criteria for compound progress. For 3CL^pro^, we used batch sizes of 250 and 500 molecules, respectively. For TNKS2 we used 5 batch sizes: 100, 300, 500, 700, and 1000. The acquisition of compounds from each RL run was done after clustering and choosing the top binders from each cluster (cluster-greedy approach) as done by Gusev et al., 2023.^15^ In this way we promote a level of exploration which is also supported by using the de novo REINVENT prior. The clusters were generated after reducing the predictor space to a 2D representation with UMAP.^60^ As descriptors we employed RDKit fingerprints and measured their similarity using the Tanimoto distance.^61^

The clustering approach we adopt here places emphasis on diversity and to a smaller degree on representativeness (by choosing the lowest Δ*G* value per cluster) but it neglects informativeness^26^ i.e., we do not include e.g., uncertainty quantification or information gain in our GAL protocol.

### 2.4 Description of Test Targets

#### 2.4.1 3CL^pro^

C30 endopeptidase, usually referred to as 3CL^pro^ or M^pro^, is the main protease in coronaviruses and plays a vital role in the lifecycle of SARS-CoV-2 (severe acute respiratory syndrome-coronavirus-2) which is the cause of COVID-19 (coronavirus disease 2019). COVID-19 led the WHO to declare a worldwide epidemic from 30 January 2020 to 5 May 2023^15^ after the first case was confirmed in November 2019. Hundreds of millions of cases have been confirmed since then and several million deaths have been directly linked to the virus.^62^ The virus and its various mutations are still a global threat to health. Several medications such as remdesivir (Veklury), nirmatrelvir/ritonavir (Paxlovid), baricitinib (Olumiant), tocilizumab (Actemra), and molnupiravir (Lagevrio) are available to treat various stages and forms of Covid-19. However, there is still a need to find new active compounds to deal with the continuously mutating virus and future related diseases. Preparation of 3CL^pro^ for simulations is described in Section 2.2.

#### 2.4.2 Tankyrase-2

Tankyrase-2 (gene TNKS2) was taken as a further test case from a large-scale binding free energy calculation benchmark data set.^37^ TNKS2 is oncogenic and regulates various cellular processes, such as telomere maintenance, mitosis, and glucose metabolism.^63^ It was chosen to study performance of GAL for a target with a more closed and confined binding pocket, compared to 3CL^pro^. It was also selected as a suitable test-case for a target for which 27 experimentally confirmed ligands from a congeneric series were available. These were used as seed structures to initialize GAL in order to see if binding affinities could be improved further and how far these generated structures would diverge structurally from the original ligands. Preparation of TNKS2 for simulations is described above in Section 2.2.

## 3 Results

In this section, we show how GAL performance has been evaluated when applied to two different test targets, 3CL^pro^ and TNKS2, in terms of chemical space exploration, structural diversity of generated compounds, and the effect of compound training batch size on obtained results.

### 3.1 Preliminary Validation

To check the ranking power of ESMACS and docking we have carried out scoring evaluations for 1014 TNKS inhibitors with IC50 from the BindingDB database. Note that ESMACS simulations are set up and run from the docking pose. We find that Spearman’s rank correlation coefficient ρ is 0.33 and Kendall’s counterpart τ is 0.22 for ESMACS and for docking ρ = 0.08 and τ = 0.05 which demonstrates that our docking protocol cannot rank compounds for this system. ESMACS shows rather moderate ranking power in this case, but it has previously been shown that ESMACS provides for accurate ranking.^64−68^ We also point out that, generally, IC50 from different sources (BindingDB contains automatic aggregation of data from multiple publications) should not be combined^69^ for correlation. Here, however, we are primarily concerned with ranking. Errors in both experiment and simulation still play a role though. Using a simple Monte Carlo approach and assuming normally distributed standard error for ESMACS (10 replicates) and experimental IC50 error we find that ρ = 0.21 and τ = 0.14. Experimental errors for pIC50 are typically in the range of 0.1 to 0.3 log units (0.14–0.41 kcal/mol) and dependent on experimental method, laboratory, etc.^69^ while standard deviations in heterogeneous assays e.g., as compiled in ChEMBL, can be as high as 0.7 log units.^70^ We assumed an error of 1.0 kcal/mol here for demonstration purposes. Equivalent data for 3CL^pro^ is not available as the virus, including the binding site of 3CL^pro^, is constantly mutating and data sets are inconsistent. The effectiveness of the acquisition function can be tested by comparing to random selection of compounds in every AL step. In Figures S14 (3CL^pro^) and S15 (TNKS2) we show the free energy distributions for both approaches. Both AL cycles were started with the surrogate model using the 10 000 initial compounds (see Section 2.1). We clearly see that random acquisition is much less efficient in finding high-scoring compounds than the “intelligent” cluster-greedy approach.

### 3.2 3CL^pro^ Target

#### 3.2.1 Evaluation of Surrogate Model

Initial training of the surrogate model based on ChemProp to predict binding affinities was achieved with a first batch of 10 000 structures whose binding free energies were calculated with ESMACS. These initial structures were selected according to their docking scores from a large library of molecules using a surrogate docking model.^34,35^ In subsequent iteration steps, those compounds that were acquired from ESMACS calculations were added to the training set and the surrogate model was trained from scratch at each step.

**Figure 2:**
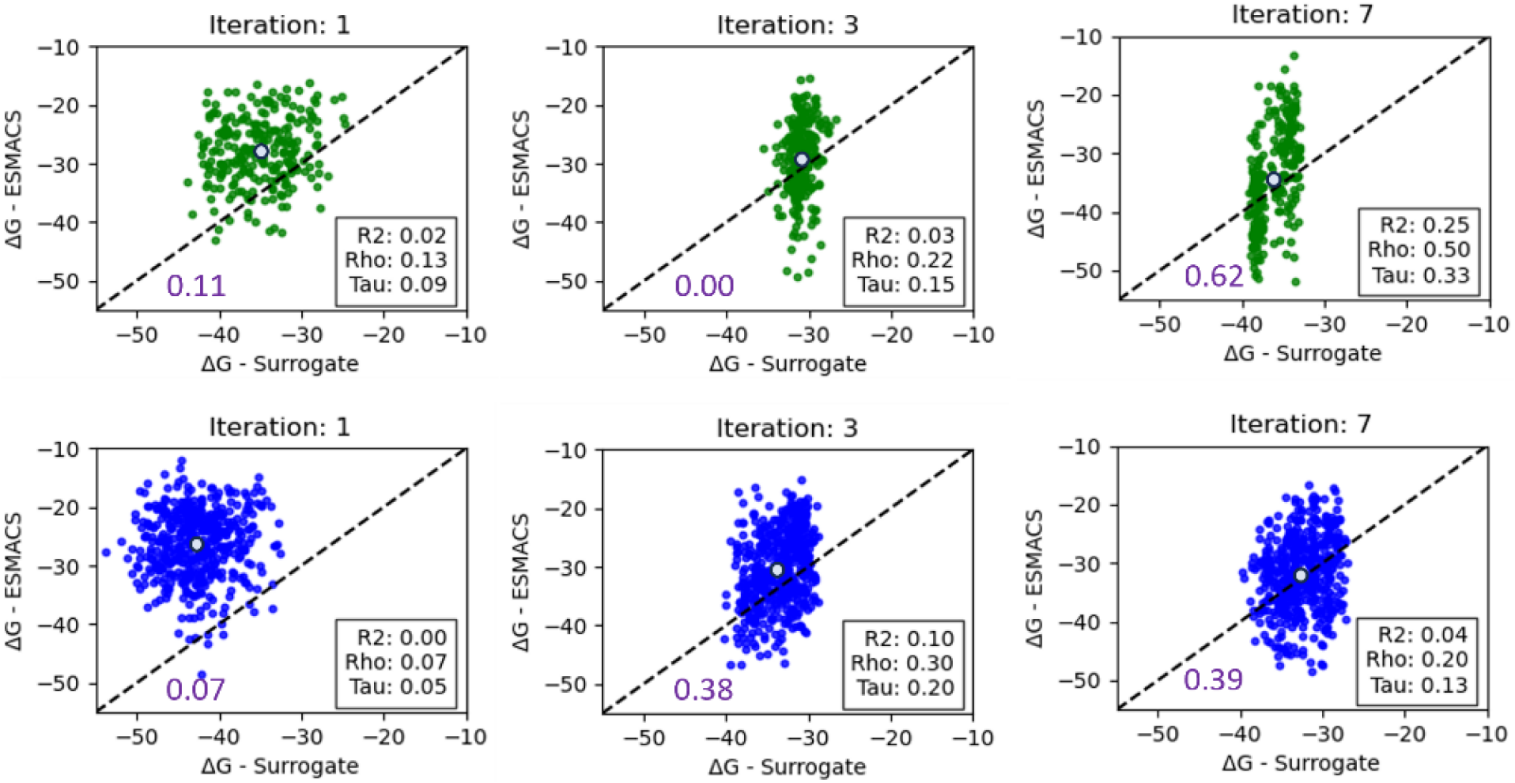
Comparison of surrogate model predictions of Δ*G*_b_ with calculated ESMACS values for training batch sizes of *n* = 250 (in green) and *n* = 500 (in blue) at selected GAL iteration steps for 3CL^pro^. *R*^2^-coefficient as well as Spearman and Kendall rank correlation coefficients rho and tau are given in the insets of each plot. The average Δ*G*_b_ of all surrogate model predictions and ESMACS calculations within an iteration is shown as a light blue circle. All energies are given in units of kcal/mol. Results for all iteration steps are given in Figures S2 and S3. The precision of the surrogate model is given in purple for each batch size and iteration step, where a true positive compound was defined here as a compound with Δ*G*_b_ < −35 kcal/mol according to the surrogate model and ESMACS prediction.

Values of the binding free energy, Δ*G*_b_, predicted by the surrogate model are compared with values derived with ESMACS, for all compounds that were acquired for oracle evaluation during GAL iterations, are shown in Figures 2, S2 and S3. Results are shown for each iteration step for small (250 molecules) and large (500 molecules) training batch sizes, respectively. The usually narrower range of Δ*G*_b_ values from the surrogate model is a result of the oracle acquisition procedure, whereby only compounds with the lowest surrogate Δ*G*_b_ values were sent to the ESMACS oracle for evaluation.

According to the results, even though the surrogate model started with a reasonable ability to rank compounds according to Δ*G*_b_ after initial training, the ability to rank newly generated compounds was found to be rather limited during the first GAL iterations. For batch size 250, this ranking ability recovered mostly toward the end of the GAL procedure, whereas recovery for batch size 500 occurred to a lesser extent.

In any event, already in the first iteration step, the surrogate model did identify numerous high-scoring molecules, according to surrogate scores, some of which were true positives. The number of these true positives, i.e., compounds with good binding affinity according to surrogate and oracle scores, steadily increased from thereon. The ratio of true positives over true and false positives, i.e., the precision of the surrogate model, also steadily improved, as shown in Figure 2 and indicated by decreasing Δ*G*_b_ values predicted with ESMACS (light blue circle) that gradually approached initially overoptimistic Δ*G*_b_ values estimated by the surrogate model. Precision values that started from 0.11 and 0.07 for smaller and larger training batch sizes, respectively, decreased after the first iteration and eventually increased to values of 0.62 and 0.39, respectively, with qualitatively similar trends for both training batch sizes. An initial deterioration of surrogate model quality is not surprising given the fixed model capacity and expected covariate shift in chemical space (*cf*. diversity analysis below) which is of principal concern in AL. Subsequent recovery of prediction quality was facilitated by structural convergence of generated structures toward the end of the AL process. It is also worth pointing out here that the surrogate model was built on compounds without stereochemistry and charge states because the REINVENT prior does not support those. Also, we did not include a scoring component for REINVENT describing the intricacies of protein–ligand interactions. REINVENT operates here with limited information relative to the ESMACS oracle which necessarily has an impact on the correlation between oracle and surrogate predictions.

In the following sections, we will see that even though the ability of the surrogate model to rank compounds is limited, the precision achieved for finding high-scoring molecules is sufficient to generate new compounds with increasingly favorable binding free energies after each iteration.

#### 3.2.2 Distribution of Binding Free Energy

**Figure 3:**
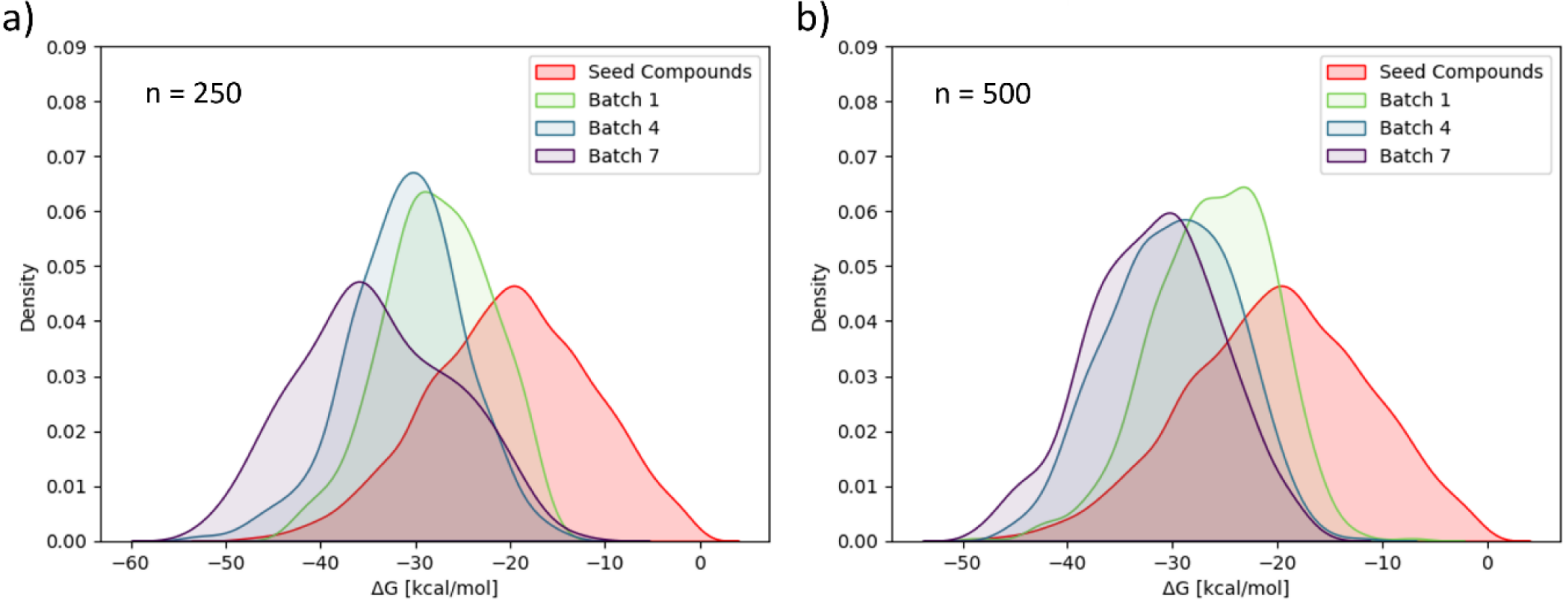
Distribution of calculated Δ*G*_ESMACS_ after a selected number of GAL iteration steps for (a) batch size 250 and for (b) batch size 500 for 3CL^pro^. The Δ*G*_ESMACS_ distribution of seed compounds used to train the initial surrogate model is shown in red. Δ*G*_ESMACS_ distributions for all iteration steps are shown in Figure S4.

The binding free energy distributions derived with ESMACS, Δ*G*_ESMACS_, are shown in Figure 3. Additionally, in Figure S5 the Δ*G*_ESMACS_ distribution of the 100 highest-scoring molecules are shown for all compounds combined after each GAL cycle.

Overall, a steady shift of energy distributions toward lower values is observed with convergent behavior toward the end of GAL. A strong enrichment of the low energy tail with more high-scoring molecules, compared to the original seed structures, is evident. For the small training batch size additionally an elongation of the lower energy tail toward lower values can be seen. The Δ*G*_ESMACS_ distributions for all acquired compounds combined in Figure S5 also show a steady shift of the 100 highest-scoring molecules toward lower binding energies until the end of the GAL run.

**Figure 4:**
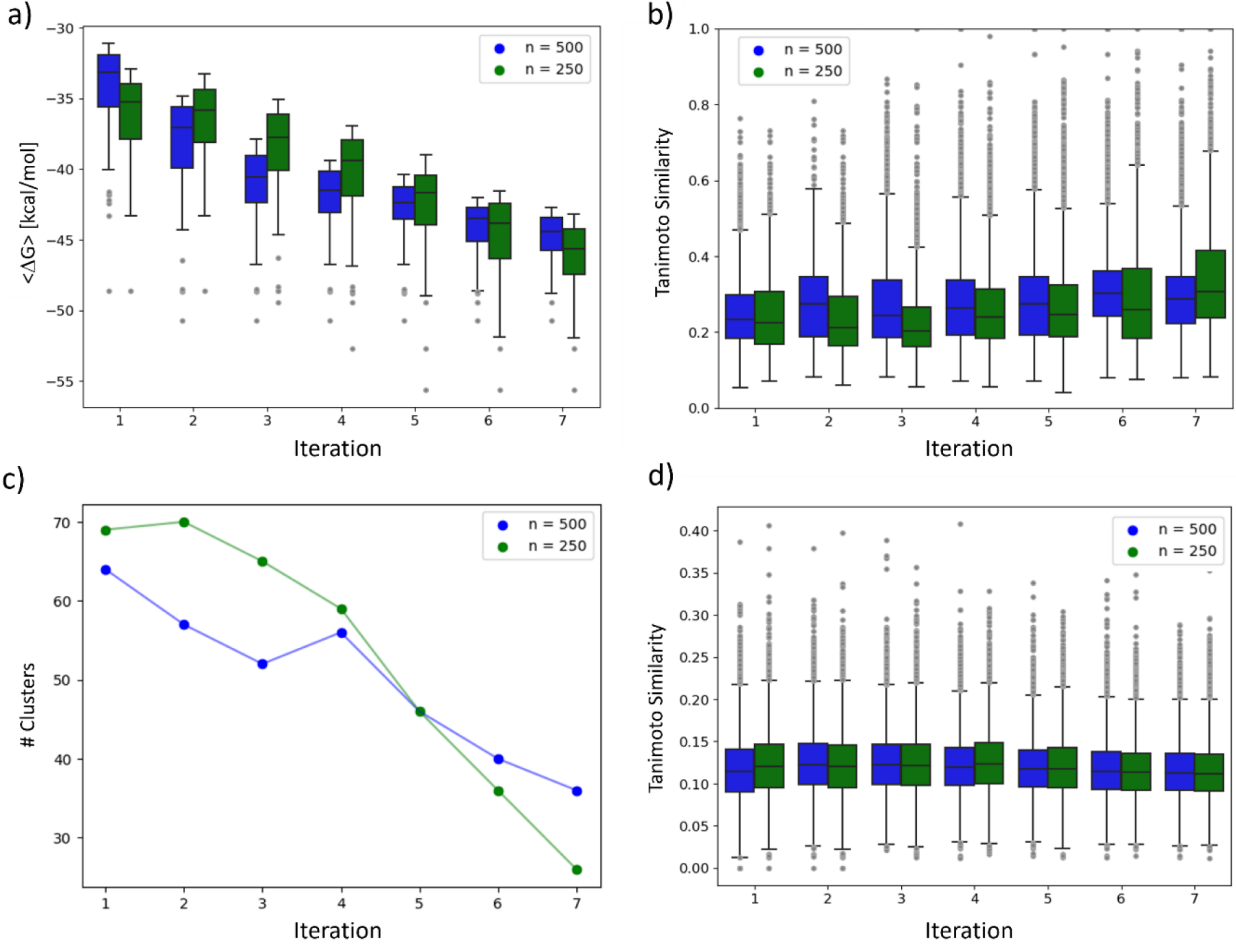
(a) Distributions of Δ*G*_ESMACS_, (b) distributions of Tanimoto similarities, and (c) number of structure clusters for each GAL iteration, for learning batch sizes 250 and 500, in green and blue, respectively, for 3CL^pro^. Also in (d) distribution of Tanimoto similarities of all pairs that contain one generated molecule and one seed compound used for the initial surrogate model training. Only the 100 compounds with lowest Δ*G*_ESMACS_ were considered and taken from the accumulated pool of compounds after each iteration.

In Figure 4a, the distribution of Δ*G*_ESMACS_ after each GAL cycle is also shown. An almost linear decrease of the average Δ*G*_ESMACS_ was obtained even though the more relevant low energy tail of the distribution, according to Figures 3 and 4a, did not move further toward lower Δ*G*_ESMACS_ during the last cycles. This means that we see an enrichment of local minima of Δ*G*_ESMACS_ with similar structures rather than finding compounds with stronger binding affinities. A possible explanation for this behavior is that GAL converged to a local minimum in chemical space, where structural variation cannot lower Δ*G*_ESMACS_ any further. However, we also note that predicted binding free energies are below −50 kcal/mol which, in our experience, is the lower limit of ESMACS free energies. Achieving structural diversity with comparably low Δ*G*_ESMACS_ is very valuable for drug design as it increases the number of possible leads for further optimization.

Overall, the results clearly demonstrate that each GAL cycle produced more structures with strong predicted binding affinities with Δ*G*_ESMACS_ values similarly low or even up to 5 kcal/mol lower than the highest-scoring molecules from the original 10 000 seed compounds. It also appears that, after about 6–7 GAL cycles, compound generation has more or less converged, i.e., only a few new compounds with relatively lower Δ*G*_ESMACS_ are found after each cycle.

Now it might perhaps be suspected that the new structures generated are very similar to the best binders from the original seed compounds. For this reason, the structural diversity of generated compounds is analyzed in the next section.

#### 3.2.3 Structural Diversity

In Figure 4b the distribution of Tanimoto similarities of all possible molecule pairs, which is directly related to internal structure diversity of a group of compounds^71,61^, of the generated 100 highest-scoring molecules, is shown after each iteration *i*, taken from the pool of compounds accumulated throughout iterations 0 to *i.*(15) The Tanimoto similarity of two compounds is dervied from their two Morgan fingerprints^72,73^ Average Tanimoto similarities start at a very low value of about 0.25, indicating that the initially generated structures are highly diverse. This value then increases during GAL when compounds from subsequent cycles are added but generally low values <0.35 were maintained throughout. An increase in similarity during GAL is symptomatic for convergence of compound generation, where increasingly similar compounds are generated once REINVENT learns which type of compounds are high-scoring molecules. Observed similarity distributions for small and large GAL training sizes were found to be comparable.

To get a better understanding of the structural diversity in the structures generated, a cluster analysis was performed for the 100 highest-scoring molecules after each cycle using the accumulated pool of generated structures. We used the Butina clustering algorithm^74^ with a cutoff value of 0.5 Tanimoto similarity. In Figure 4c the total number of found clusters is shown. A strong decrease in the number of structural clusters was observed, meaning that the top 100 binders could be grouped into fewer structural clusters with each GAL cycle. This finding is in line with the previously observed enrichment of the low energy tail of Δ*G*_ESMACS_ as can be seen in Figure S5.

In Figure S6, the population size of each found cluster together with its corresponding average Δ*G*_b_ value is shown after a different number of GAL cycles, again for the 100 highest-scoring molecules of the accumulated structures. During the GAL run, the number of structure clusters decreases, as already seen before, whereas the spread of compounds into clusters narrowed so that eventually only a few clusters were eventually significantly populated. Each cluster can be seen as a region in chemical space in which Δ*G*_ESMACS_ has a local minimum. The GAL process increasingly populates these regions in chemical space with more structures, while abandoning other local minima with higher Δ*G*_ESMACS_.

**Figure 5:**
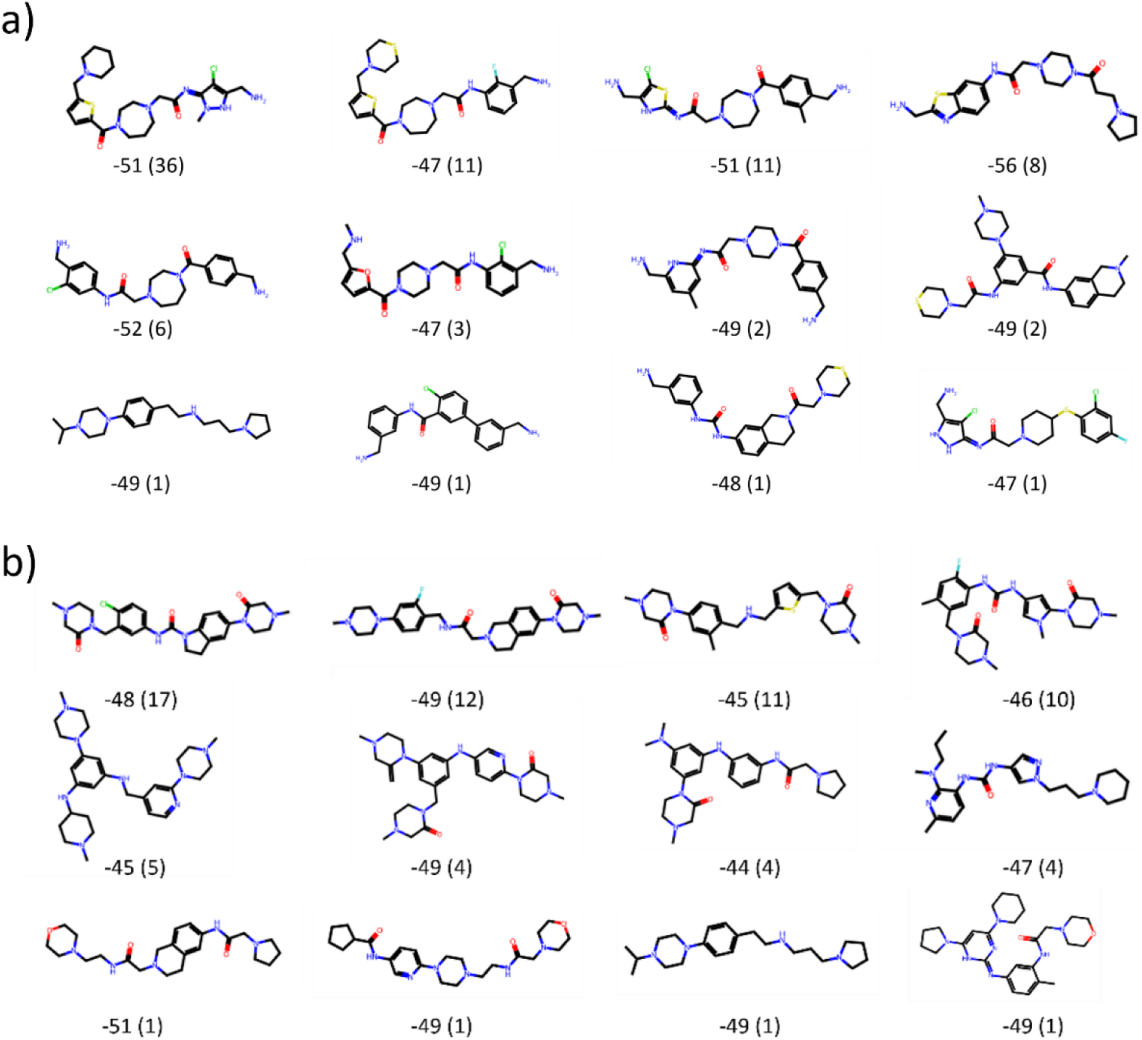
Representative chemical structures with lowest Δ*G*_ESMAC_, for different selected structural clusters from 3CL^pro^. The eight most populated clusters were chosen as well as four further clusters with lowest Δ*G*_ESMACS_. Cluster analysis was performed for those 100 compounds with lowest Δ*G*_ESMACS_ taken from the accumulated pool of compounds after each iteration for GAL training batch sizes (a) *n* = 250 and (b) *n* = 500. The energies are given in units of kcal/mol, the cluster population sizes are given in parentheses.

To get a better understanding of the structural similarity within a cluster and between different clusters, representative chemical structures are displayed in Figure 5. For some selected clusters the structure with lowest Δ*G*_ESMACS_ is displayed. Population size of each cluster is given in parentheses. We can see the Butina clustering generated clusters that contain structures with varying scaffolds, even though various structural motives occur frequently. Overall, a diverse set of high-scoring molecules with different scaffolds were obtained with our GAL protocol.

The question next arises as to how similar the compounds found are to the original seed compounds that were used to train the surrogate model. Is our GAL protocol able to find genuinely new compounds or only variations of what was used to seed the process? To answer this question, the Tanimoto similarity distribution of all possible pairs that contain one of the 100 generated highest-scoring molecules and one of the original seed compounds was evaluated and is displayed in Figure 4d.

**Figure 6:**
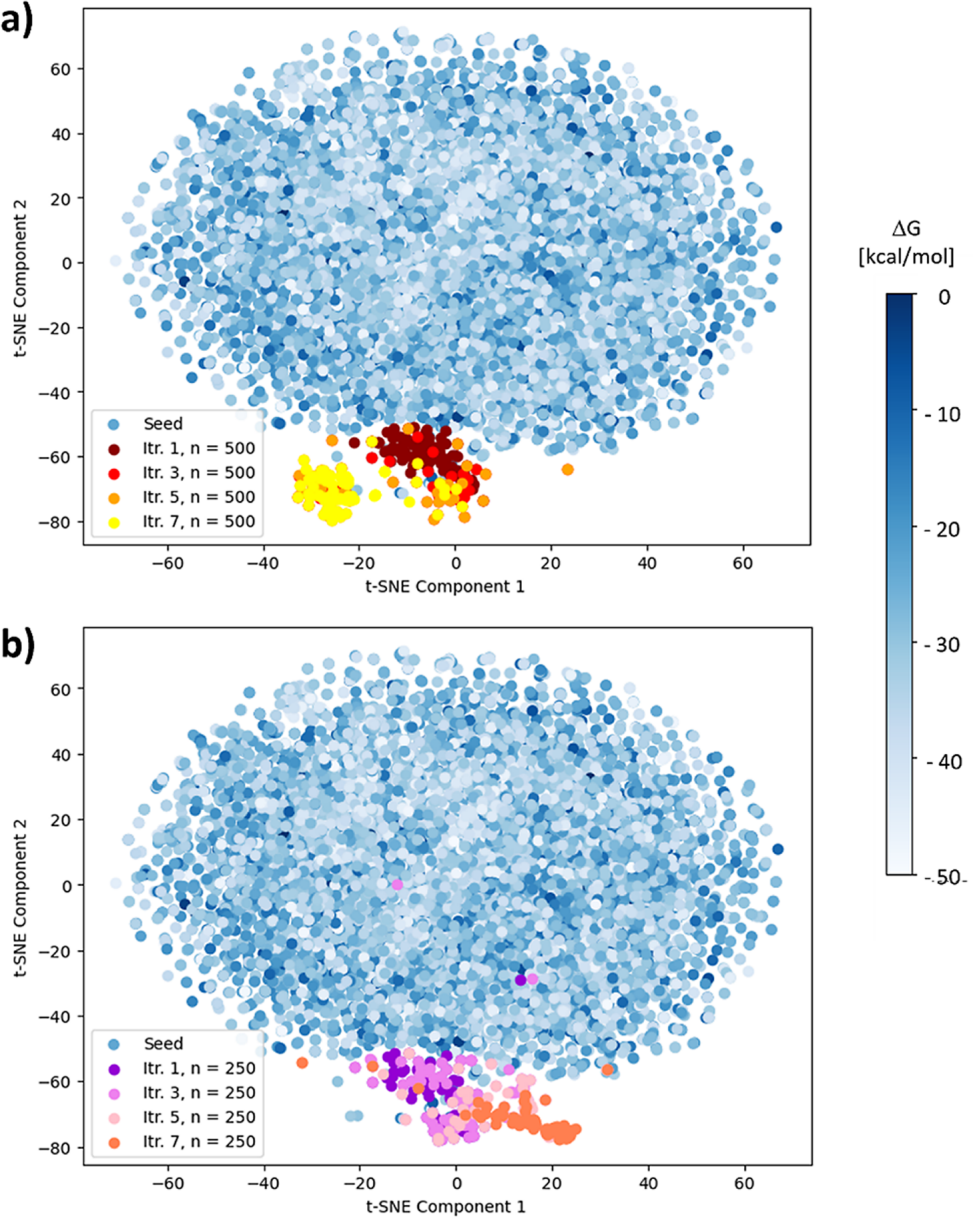
Morgan fingerprints of compounds projected into 2D using t-SNE, calculated for all seed compounds and generated compounds from large and small training batch sizes combined for 3CL^pro^. Seed compounds used for initial training of the surrogate model are shown in shades of blue according to calculated Δ*G*_ESMACS_, color coded as shown in the legend on the right side. Deviations of newly generated molecules from seed compounds are shown for GAL training batch sizes (a) *n* = 250 and (b) *n* = 500 for some selected iterations, color coded as shown in the legend insets. Molecules were taken from those 100 compounds with lowest Δ*G*_ESMACS_ from the accumulated pool of compounds after each iteration.

Very low average Tanimoto similarities of compounds generated in GAL cycles and seed compounds below 0.13 were observed. Values of the most extreme outliers decrease somewhat from about 0.4 to 0.35 throughout the GAL process. This demonstrates that the generated highest-scoring molecules are indeed genuinely new compounds and are structurally dissimilar from the original seed compounds. Furthermore, the decreasing value of maximum similarity in Figure 4d also suggests that, during GAL, the generated compounds drift away from the chemical space occupied by the seed compounds. To highlight this crucial aspect of GAL further, we visualize occupancy and drift of chemical space with dimensionality reduction in Figure 6.

We used 2D t-distributed stochastic neighbor embedding^75^ (t-SNE) of the Morgan fingerprints of the 100 generated highest-scoring molecules as well as of the original seed compounds to visualize movement in chemical space throughout the AL process in Figure 6. Data points in blue represent the region of chemical space occupied by the original seed compounds. We found that newly generated compounds substantially deviated from the chemical space region of the seed compounds already after the first iteration. Moreover, we also see that generated structures of high-scoring molecules cover a smaller chemical space than the seed compounds, which is expected as the majority of the very diverse seed compounds also cover a wide range of binding affinities. It appears that, after about five learning cycles, GAL structures of the small and large training batch sizes moved into similar chemical space regions that are well away from the seed structure chemical space, but successively diverged into two distinct chemical space regions. In other words, the two GAL runs converged to two different local Δ*G*_ESMACS_ minima in chemical space. This is not necessarily caused by the different batch sizes but more likely reflects the nondeterministic nature of the GAL protocol, which involves use of various random seeds and RL using multinomial sampling to draw samples. Most importantly, these results clearly demonstrate that the generated structures of high-scoring molecules are genuinely new compounds and not just mere variations of some of the seed compounds.

Overall, we demonstrated that the AL process found genuinely new and diverse compounds with varying scaffolds and strong binding affinities exceeding those of the highest-scoring molecules of the original seed compounds. Moreover, after initial training of the surrogate model with 10 000 compounds, this was achieved after only 7 iteration steps with a total of 3500 and 1750 oracle calls, for large and small training batch sizes, respectively.

#### 3.2.4 Diversity of Ligand Binding Modes

**Figure 7:**
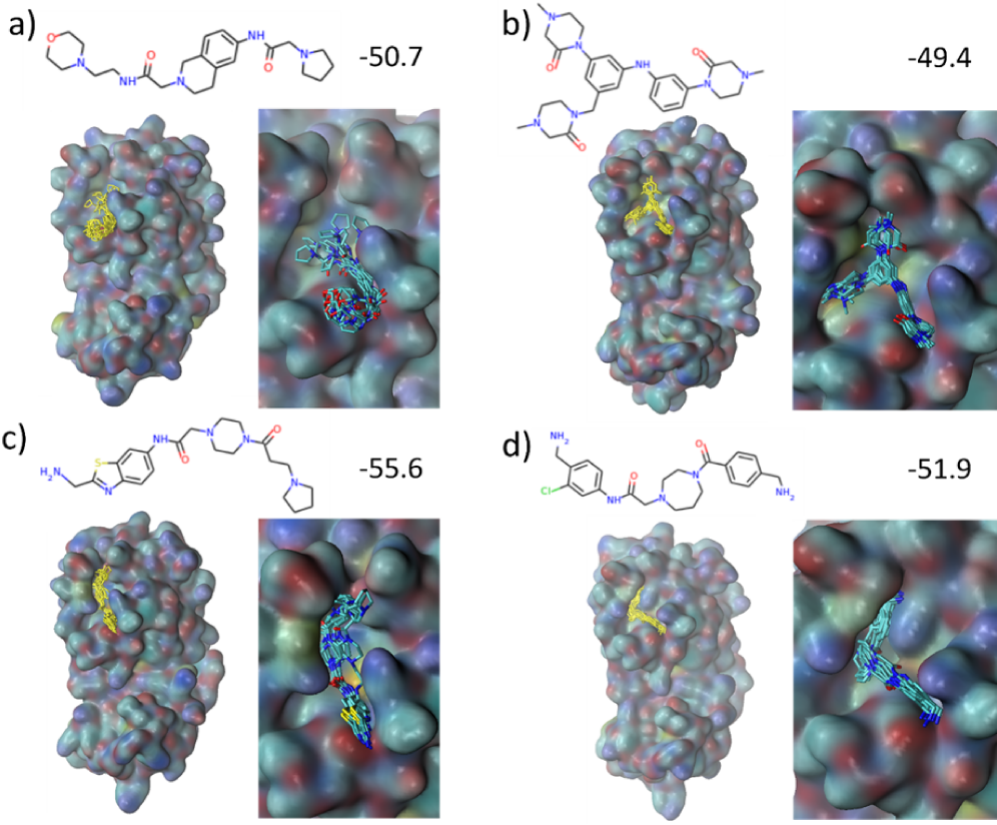
(a–d) Four selected ligands with predicted high target binding affinity in the 3CL^pro^ binding pocket. Molecules in (a,b) and (c,d) were taken from training batch sizes 500 and 250, respectively. Chemical structures together with their binding free energy Δ*G*_ESMACS_ (in units of kcal/mol) are given on top of the figures. Shown ligand structures, highlighted in yellow on the left-hand sides, are superpositions of snapshots taken from ten ESMACS replicas after 4 ns of MD simulation. Protein surfaces are shown with coloring according to atom types of the surface atoms. The ligands together with their protein vicinity are shown in more detail on the right-hand side for each ligand.

In Figure 7 we investigated the binding modes of four selected ligands as examples for binding to the 3CL^pro^ target protein. These four ligands were generated during the GAL run and found to exhibit strong binding affinities with −56 kcal/mol < Δ*G*_b_ < −49 kcal/mol as well as low chemical similarity among each other.

We found different binding modes for these four ligands as demonstrated by different contact areas between ligand and protein and hence substantial differences in ligand–protein interactions. A closer inspection of these complexes showed the importance of hydrogen bonds between ligand amines, several of which are found in all generated binders with strong binding affinity, and the protein, whereas ligand carbonyl groups seem much less engaged in hydrogen bonding. Amines formed hydrogen bonds inconsistently with different protein residues when comparing across ligands. Only hydrogen bonds with anionic Glu166 and Thr25 were obtained consistently. Moreover, we found that ligands interact with different parts of the active site, where the additional ring group of the only nonlinear compound shown in Figure 7c provides specific binding to an active site region that cannot be reached by the linear compounds.

A more comprehensive analysis of binding modes is beyond the scope of the current study. However, overall, observed binding modes exhibit additional diversity that could potentially be exploited in a subsequent study by combining moieties found to interact with different sections of the binding pocket.

#### 3.2.5 Computational Efficiency

Comparing the quality of GAL compounds generated with different training batch sizes, as discussed in this work, the question arises which batch size is the computationally most efficient. The computationally most demanding step by far in the GAL workflow is the derivation of ESMACS Δ*G*_b_ values for each oracle call. While larger batch sizes require more computational effort, fewer iterations are needed to find high-scoring molecules as discussed before. This behavior could favor larger batch sizes where many compute nodes are available to run in parallel; however, when that is not the case, smaller batch sizes might be more efficient.

To investigate this further, we define computational efficiency η as the number of suitable structures found per made oracle call:
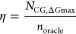


The number of oracle calls *n*_oracle_ is then simply the training batch size multiplied by the number of iterations. Defining when a generated compound is considered as suitable is less straightforward. Generally, a good compound should exhibit a large binding affinity and it should be sufficiently different in structure from other compounds generated so far. We defined compounds as suitable when their binding free energy Δ*G*_b_ < Δ*G*_max_, where different values for the threshold Δ*G*_max_ were considered. Additionally, a cluster analysis was performed, using the Butina algorithm, for those structures with Δ*G*_b_ < Δ*G*_max_, and counted the number of found clusters as the number of compound groups sufficiently different form each other, *N*_CG_. The similarity cutoff of the clustering algorithm, *s*_cutoff_, is the similarity required for two structures to belong to the same cluster. A larger value of *s*_cutoff_ therefore means that only very similar compounds are grouped into the same cluster and means that larger and fewer clusters are generated, while individual clusters cover a larger area in chemical space.

The situation where a small value of *s*_cutoff_ is used, i.e., where compounds are grouped together into more clusters with fewer cluster members, together with a more demanding Δ*G*_max_ = −40 kcal/mol corresponds to a situation where compounds are sought that exhibit a high binding affinity but where structural diversity is less important as essentially all newly generated compounds are counted toward η, regardless of how similar they are to already generated structures. This is a typical situation akin to lead optimization in drug discovery. In contrast, in case of a more relaxed Δ*G*_max_ = −35 kcal/mol and larger *s*_cutoff_, structural diversity and coverage of chemical space is more emphasized than binding affinity, which is typical for a more explorative hit finding scenario. Therefore, through selection of Δ*G*_max_ and *s*_cutoff_ we can evaluate GAL effectiveness for these different situations.

**Figure 8:**
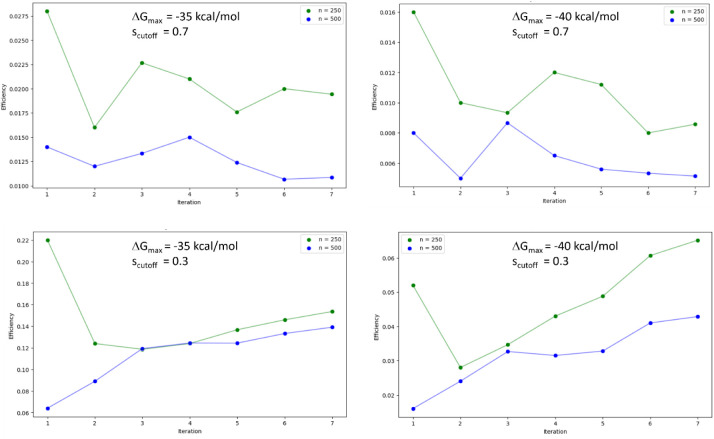
Efficiency of GAL for the 3CL^pro^ target using two different training batch sizes after each iteration step. Efficiency is defined as the number of structural clusters found per oracle call of all cumulatively generated structures at a given iteration step. Only ligands with Δ*G*_b_ < Δ*G*_max_ were considered. Clustering was carried out using the Butina algorithm with a similarity cutoff given in respective plots.

The results for η are shown in Figure 8 for the two different training batch sizes and two different values for Δ*G*_max_ and *s*_cutoff_ throughout the GAL for 3CL^pro^. Δ*G*_max_ = −35 kcal/mol corresponds approximately to the maximum of the Δ*G* distribution obtained after the last GAL iteration (see Figure 3), whereas Δ*G*_max_ = −40 kcal/mol represents a stricter criterion to define suitable structures. In all cases, the smaller training batch size of *n* = 250 was found to be computationally more efficient. For *s*_cutoff_ = 0.3 improvements in efficiency were observed with increasing iteration steps, whereas for *s*_cutoff_ = 0.7 efficiency remained more or less the same throughout the GAL run.

### 3.3 Tankyrase-2 Target

#### 3.3.1 Generation of First Compound Batch

The starting point of GAL for the tankyrase-2 (TNKS2) target was a congeneric series of 27 ligands with experimentally confirmed favorable binding free energies with a range from −35 to −26 kcal/mol as computed with ESMACS. Binding affinities derived with ESMACS were also compared to measured affinities, as shown in Figure S7, where a reasonable ranking of compounds was obtained through simulations. We then proceeded to generate the first training batch of compounds with the method described in Section 2.1. In the following, we will refer to the GAL step using the initially generated compounds as iteration zero. Five GAL steps were carried out with variations only in the training batch size, using sizes *n* = 100, 300, 500, 700, and 1000 compounds per iteration. Overall, we found that only four iterations were sufficient for results to converge, as shown below.

#### 3.3.2 Quality of Surrogate Model

**Figure 9:**
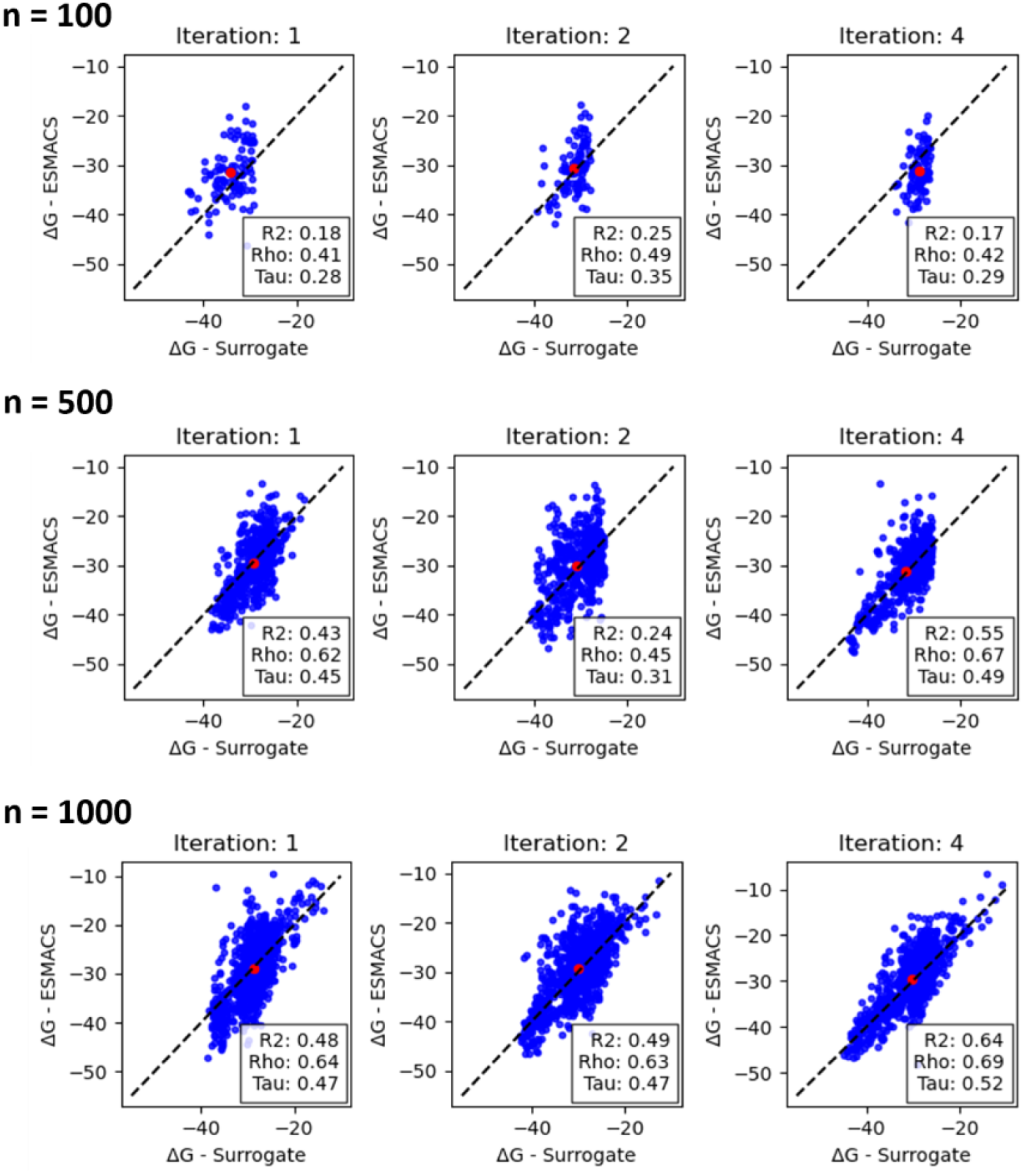
Comparison of surrogate model predictions of Δ*G*_b_ with calculated ESMACS values for training batch sizes between 100, 500, and 1000 molecules for selected GAL iteration steps for TNKS2. *R*^2^-coefficient as well as Spearman and Kendall rank correlation coefficients rho and tau are given in the insets of each plot. The average Δ*G*_b_ of all surrogate model predictions and ESMACS calculations within an iteration is shown as a red circle. All energies are given in units of kcal/mol. Results for all training batch sizes and iteration steps are shown in Figure S8.

In Figure 9 binding affinity predictions of the ChemProp surrogate model are compared with Δ*G* derived with ESMACS for a selection of batch sizes and iteration steps (Figure S8 contains this data for the entire data set). First, we obtained a substantially improved quality of the surrogate model predictions compared to 3CL^pro^. Large values for Spearman ranking and *R*^2^ coefficients above 0.7 and 0.6, respectively, were obtained. A comparison across batch sizes shows that reliable surrogate models were found after iteration 3 for all sizes, except to a lesser extent for the smallest training batch size of *n* = 100. We also observe that for *n* = 500 and larger, good surrogate models were found already after the first iteration step. For GAL it means that far more true positives, i.e., compounds with favorable binding affinities according to ESMACS, were found and a higher precision was achieved. How this affected GAL overall will be described in the following. Possible reasons behind the better surrogate model quality as compared to 3CL^pro^ are discussed in Section 3.3.5.

#### 3.3.3 Δ*G*_b_ Distribution

**Figure 10:**
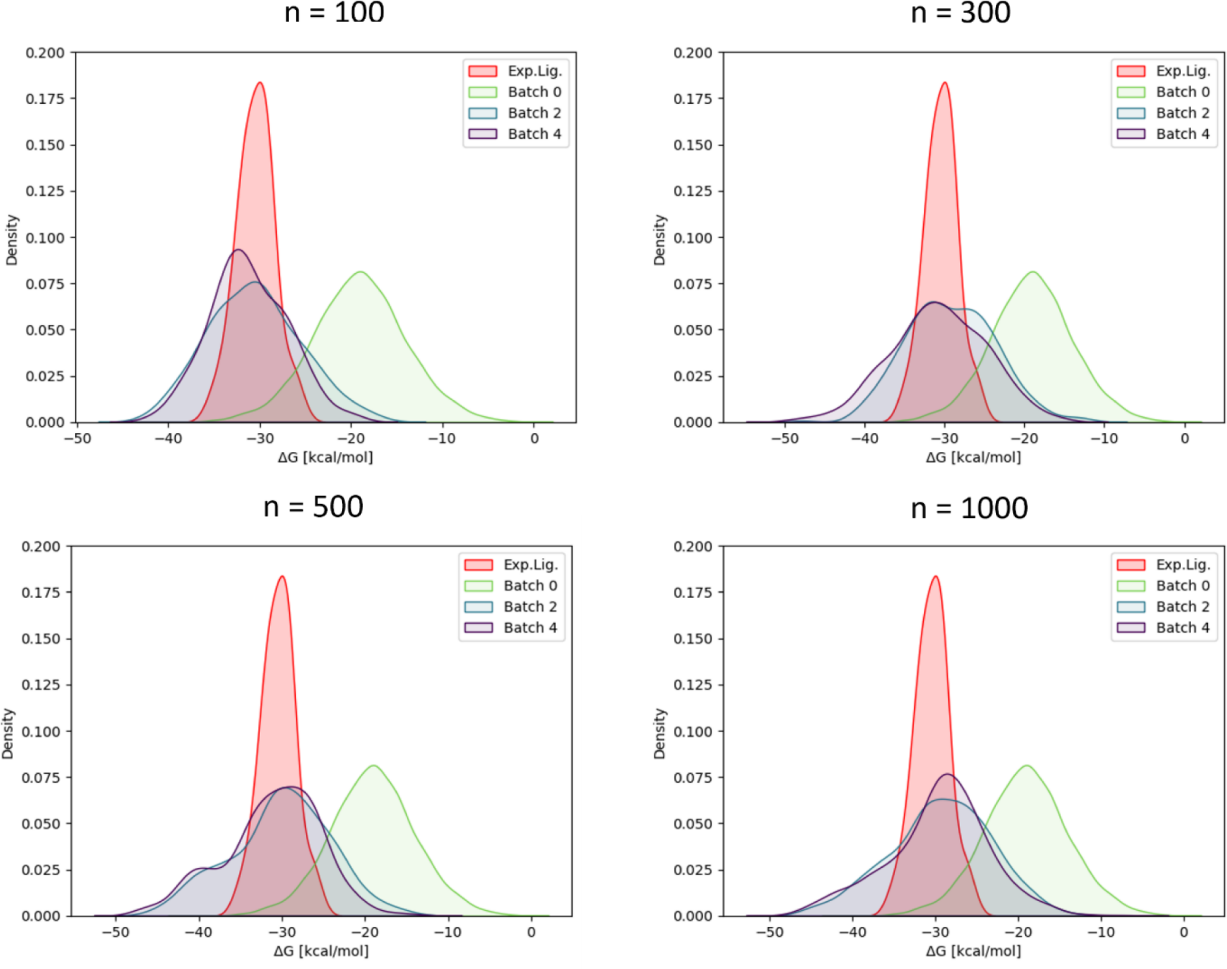
Distribution of calculated Δ*G*_ESMACS_ for selected GAL iterations using different batch sizes for TNKS2. The Δ*G*_ESMACS_ distribution of 10k seed compounds used to train the initial surrogate model is shown in green as batch 0. The Δ*G*_b_ distribution of 27 measured compounds is shown for comparison in red. Results for all batch sizes and iteration steps are shown in Figure S9.

In Figure 10, distributions of obtained Δ*G*_b_ values for selected iteration steps are shown (full data set included in Figure S9). For comparison, the measured values of the congeneric series of the original 27 ligands are also shown together with the Δ*G*_b_ distribution of the compounds from iteration zero. We found that, already after the first iteration, substantial improvements were achieved compared to the initial iteration zero compound sample and that the high affinity tail of the distribution even extended beyond the binding energies of the experimentally confirmed ligands, which are already characterized by strong binding affinities. Moreover, we found that for *n* = 500 and larger, iterations after step 1 barely improved the energy distribution further, whereas for the smaller batch size of *n* = 300 improvements were still achieved with additional iterations. For *n* = 100 not much improvement was obtained with more iterations and the high affinity tail was less extended toward lower energies than it was the case for the other batch sizes.

**Figure 11:**
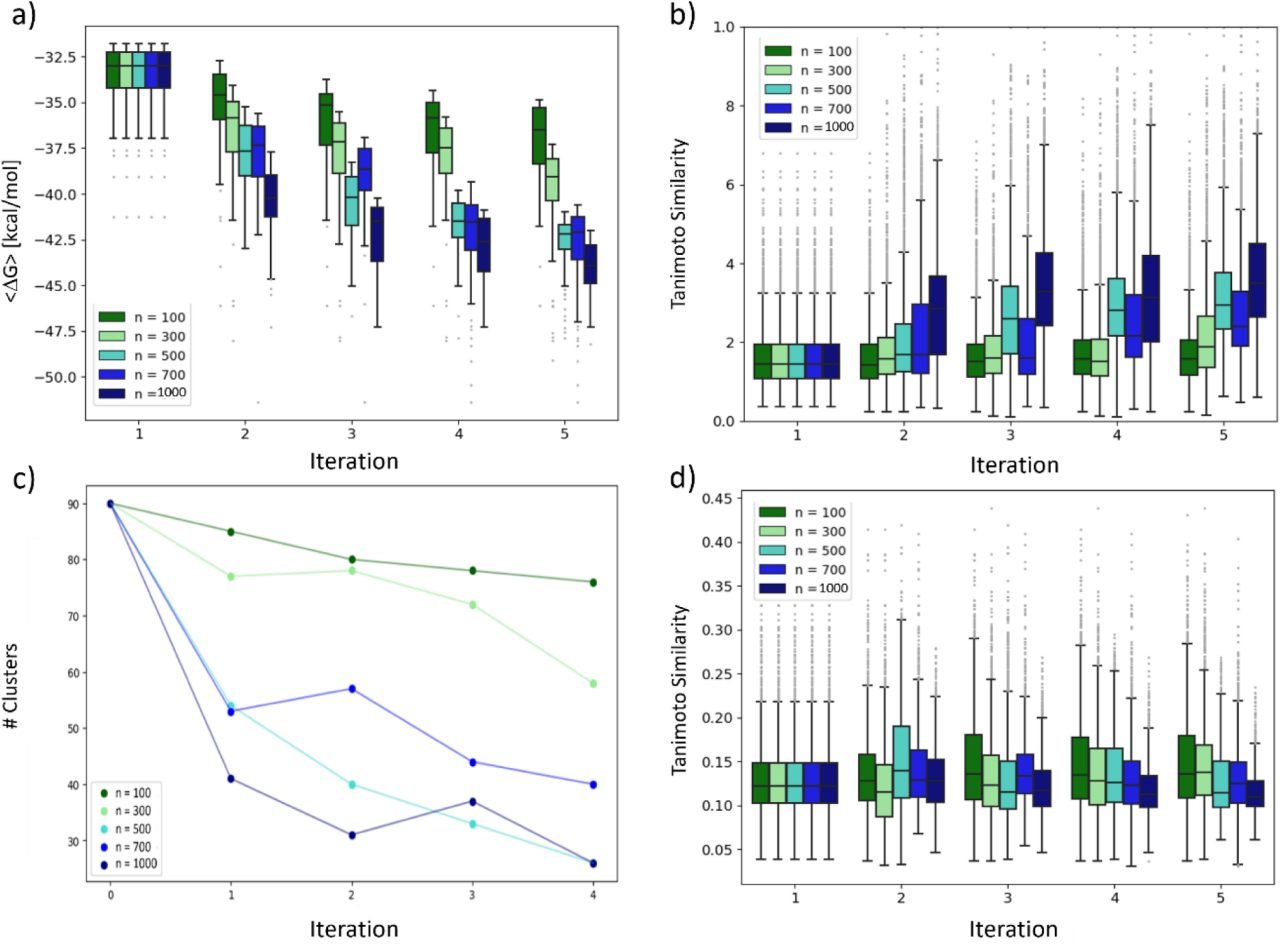
(a) Distributions of Δ*G*_ESMACS_, (b) distributions of Tanimoto similarities, and (c) number of structure clusters for each GAL iteration and for different learning batch sizes used for TNKS2.

Overall, convergence of Δ*G*_b_ distributions was basically achieved rapidly within a single iteration (except for *n* = 300), compared to five or more iterations for 3CL^pro^. In Figure 11a changes in the Δ*G*_b_ distribution throughout the GAL run are shown for the 100 highest-scoring molecules of the cumulatively generated compounds: there is a trend to lower values of <Δ*G*_b_> for larger batch sizes and a steady decrease with an increasing number of iteration steps. Here, larger batch sizes enable the development of more precise surrogate models as shown before, facilitating moderate improvements in <Δ*G*_b_> that, however, come with the computational cost of more oracle calls. The computational efficiency of GAL is discussed in Section 3.3.6.

Also in (d) distribution of Tanimoto similarities of all pairs that contain one generated molecule and one of the 27 molecules from the congeneric series used to generate the first traning batch of compounds. Only the 100 compounds with lowest Δ*G*_ESMACS_ were considered and taken from the accumulated pool of compounds after each iteration.

We also explored if structural diversity could be improved further by augmenting the surrogate model with known TNKS2 IC_50_ values from BindingDB.^24^ Structures having the same core as the 27 benchmark compounds and IC_50_ ≥ 1 μm were removed leaving 484 ligands. From these we randomly chose 20 compounds and added to those 80 compounds from iteration 3 to resume GAL with a training batch size of 100. Resulting Δ*G*_b_ distributions are shown in Figure S11. We found that, following minor improvements of the Δ*G*_b_ distribution after iteration step 2, no improvements were obtained after 5 iterations compared to GAL without any BindingDB infusions.

The quality of the surrogate model trained with the BindingDB infused compound samples is shown in Figure S12. The results are comparable to the previously trained surrogate models shown in Figure 9. Average Tanimoto similarities between 0.16 and 0.15 were obtained, which were essentially the same as what was previously observed for GAL without infusions, see Figure 11b. Overall, it appears that the infusion of additional structures from BindingDB did not improve the quality of GAL results.

#### 3.3.4 Structural Diversity

Internal structural diversity of generated structures is shown in Figure 11b. Generally, diversity of structures tends to be even larger than for 3CL^pro^. The average Tanimoto similarity increased from iteration to iteration, except for training batch size *n* = 100, suggesting convergence in chemical space. Less diversity was obtained in larger training batch sizes, where it can be assumed that a more exhaustive sampling of chemical space near minima of Δ*G*_b_ led to more similar structures.

In Figure 11c, the number of structure clusters according to the Butina algorithm with a cutoff of 0.5 in Tanimoto similarity is shown for the 100 highest-scoring molecules from the cumulatively generated structures. Also, in Figure S10 the average Δ*G* for each structural cluster is shown together with its population size. As for 3CL^pro^, we see that the number of clusters substantially decreased during the GAL process as clusters with structures associated with low Δ*G*_b_ energy minima were increasingly populated at the expense of clusters related to higher Δ*G*_b_ minima. Also, GAL performed with larger batch sizes led to a smaller number of clusters, which once again is related to a more pronounced convergence in chemical space.

**Figure 12:**
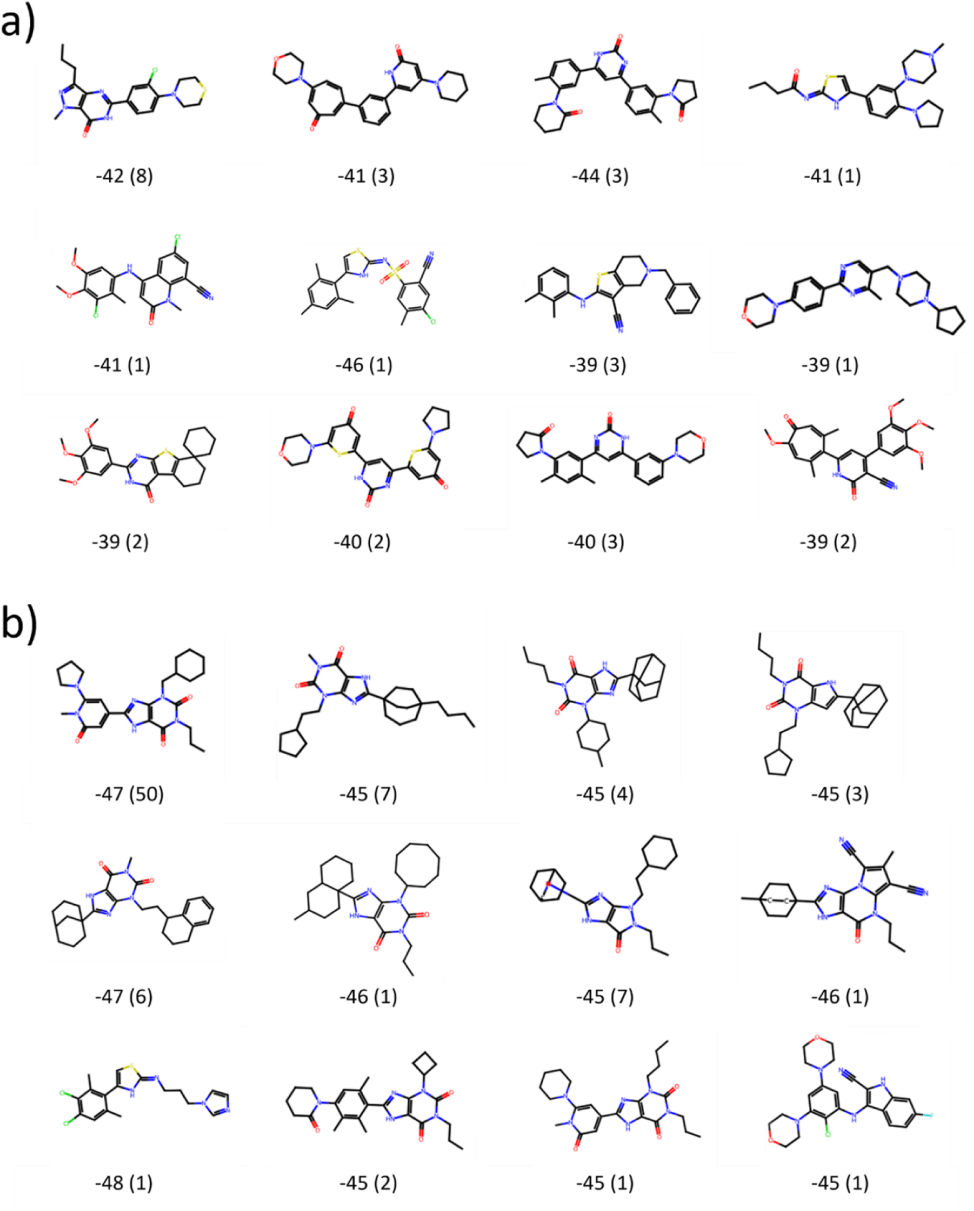
Chemical structure representatives with lowest Δ*G*_ESMAC_ for different selected structural clusters from TNKS2. The eight most populated clusters were chosen as well as four further clusters with lowest Δ*G*_ESMACS_. Cluster analysis was performed for those 100 compounds with lowest Δ*G*_ESMACS_ taken from the accumulated pool of compounds after each iteration for GAL training batch sizes (a) *n* = 100 and (b) *n* = 1000. Cluster population sizes are given in parentheses. Energies are given in units of kcal/mol.

Some structures of generated compounds are exhibited in Figure 12; this demonstrates that generated compounds with favorable binding affinities involve a large variety of different scaffolds. This is particularly noteworthy considering that the initial sample of generated compounds was based solely on a small congeneric series sharing the same quinazolinone scaffold. However, we also find quinazolinone and similar scaffolds (see Figure 12) frequently in the predicted binders. When directly compared to the congeneric series, GAL generated structures indeed manifest low Tanimoto similarities with those 27 ligands, with the majority of values found below 0.3 as shown in Figure 11d. We also observe that similarity distributions for larger training batch sizes stretch less toward larger values, i.e., more divergence from the original molecules was obtained when larger traning sizes for GAL were used.

This trend is in line with the decreasing average Δ*G*_b_ values for larger training sizes, corresponding to a further progression toward convergence in chemical space, which led to inclusion of functional groups for *n* = 1000 such as nitriles and bridged cycles.

In Section 3.3.5 we will show how these groups contribute to a minimum in Δ*G*_b_. Apart from improvements of Δ*G*_b_ we also observed somewhat higher QED scores, i.e., more drug-likeness, for larger batch sizes as shown in Figure S14, despite seeing in Figure 12b also some more uncommon compound moieties that are not found in typical drugs.

At this point, it is important to note that many generated compounds with favorable ESMACS binding affinity Δ*G*_b_ and some drug-likeness as per QED would otherwise unlikely be chosen for progression in a drug discovery project, as they may be too difficult to synthesize or lack other properties. Improvement on those issues is beyond the scope of the present work.

**Figure 13:**
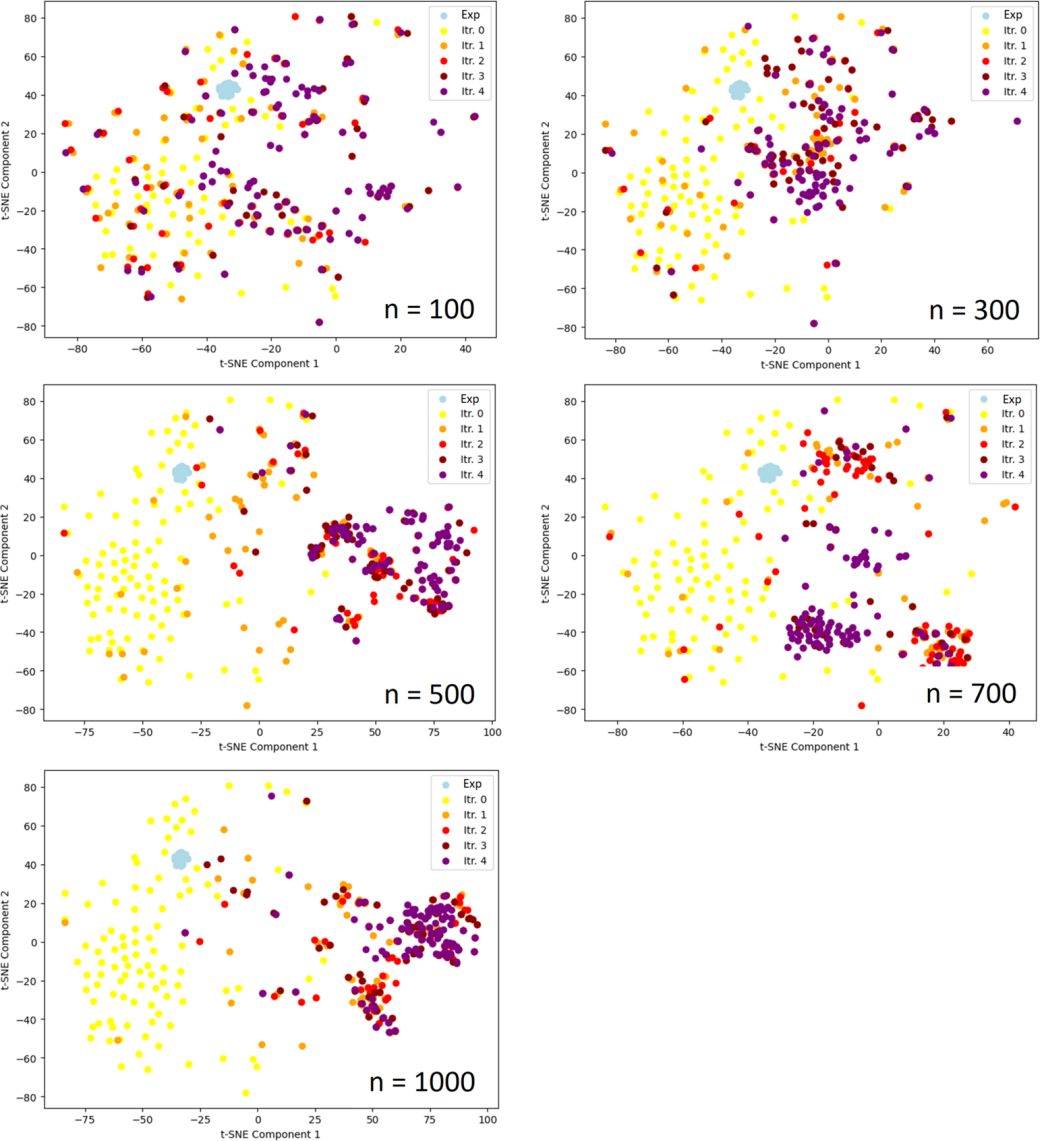
Morgan fingerprints for TNKS2 compounds projected into 2D space using t-SNE computed on the combined data set from all batches shown here to enable comparison of chemical space. Compounds from iteration 0, shown in yellow, refer to molecules taken from the 10 000 initial compounds. The 27 ligands with measured binding free energies are also included and are shown in light blue. Generated molecules are color coded as per the legend insets. The molecules shown were taken from those 100 compounds with lowest Δ*G*_ESMACS_ from the accumulated pool of compounds after each iteration.

In Figure 13 2D t-SNE visualizes the chemical space traversed throughout GAL for different training batch sizes. We observe that the original 27 ligands, shown as light-blue data points, are focused within a very small area, whereas the 10k generated structures, shown in yellow, cover a much larger chemical space. Throughout the GAL process, convergence into chemical spaces distinct from the space covered by the structures from iteration 0 is observed, which is more pronounced for GAL with larger compound training sizes. For *n* = 100 and 300, it appears that convergence did not yet occur. Furthermore, we observe that convergence was achieved for *n* = 500 and *n* = 1000 to a similar space region, whereas for *n* = 700 GAL converged to a somewhat different region.

Overall, the findings reported in this subsection indicate that GAL can generate structures with high internal diversity, well distinct from the original 27 ligands. The results also suggest that for training sizes of *n* ≥ 500, according to results shown in Figures 13 and S10, the overall 100 best binding compounds generated after the last GAL iteration could be grouped into fewer structural clusters with lower Δ*G*_b_, i.e., the generated highest-scoring molecules compounds occupied narrower regions in chemical space. It also shows that GAL with smaller batch sizes is likely to benefit from more iteration steps for structural convergence and Δ*G*_b_ optimization.

#### 3.3.5 Diversity of Binding Modes

**Figure 14:**
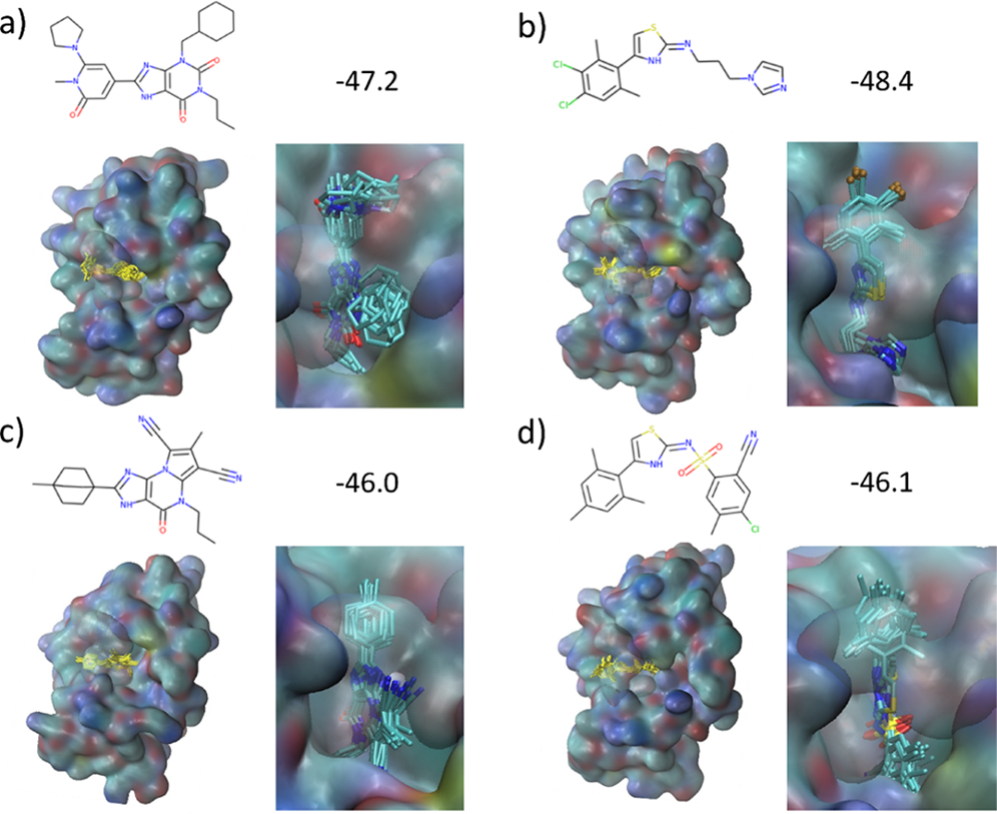
(a–d) Four selected ligands with predicted high binding affinity in the TNKS2 binding pocket. Molecules were taken from the largest and smallest training batch sizes, 1000 a,b) and 100 c,d), respectively. Chemical structures together with their binding free energy Δ*G*_ESMACS_ (in units of kcal/mol) are given above the images. The ligand structures shown, highlighted in yellow at the left-hand side of each image, are superpositions of snapshots taken from the ten ESMACS replicas per ensemble after 4 ns of MD simulation. Protein surfaces are shown with coloring according to atom type of the surface atoms. The ligands together with their protein vicinity are shown in more detail on the right-hand side for each ligand.

Four representative, low Δ*G*_b_ structures bound to TNKS2 are shown in Figure 14. Compared to 3CL^pro^ a qualitative difference is apparent: in 3CL^pro^ a variety of different binding modes was found due to the large open binding pocket of the target, whereas in the case of TNKS2 the binding pocket is narrow and closed, which led to a well-defined binding mode and far less conformational variation of ligands in the binding pocket, despite large structural differences across ligands. A closer inspection of the ligand–protein interface reveals that some of the moieties improving Δ*G*_b_ were frequently found within the generated structures. For instance, nitriles were included into ligands to optimize the distance between the ligand and the protein surface through the triple bond. Bridged bicyclic moieties turned out to act as bespoke “plugs” that close the opening of the active site on one side, thereby maximizing van der Waals interactions with the protein, which would have been otherwise difficult to achieve with flat rings or other groups. These are instructive examples showing how REINVENT optimized the design of compounds in GAL to fit the active site of a protein target.

It is reasonable to assume that the confined space in the active site of TNKS2 and the correspondingly well-defined binding pocket structure permit a less ambiguous correspondence of the ligand structure, represented in REINVENT as a 1D SMILES code, and the 3D docking pose of the ligand inside that active site and thereby binding free energy. This could have facilitated the development of a more predictive surrogate model for GAL than in the case of 3CL^pro^ (given that our surrogate model only considers ligand structures but not those of the protein, thereby does not capture protein–ligand interactions explicitly), in turn enabling the discovery of new structures with good binding affinities within a smaller number of GAL steps. This indicates that performance of the GAL process, even though successful for both selected test targets, can be dependent on the extent of ligand confinement in the active site of the target.

#### 3.3.6 Computational Efficiency

**Figure 15:**
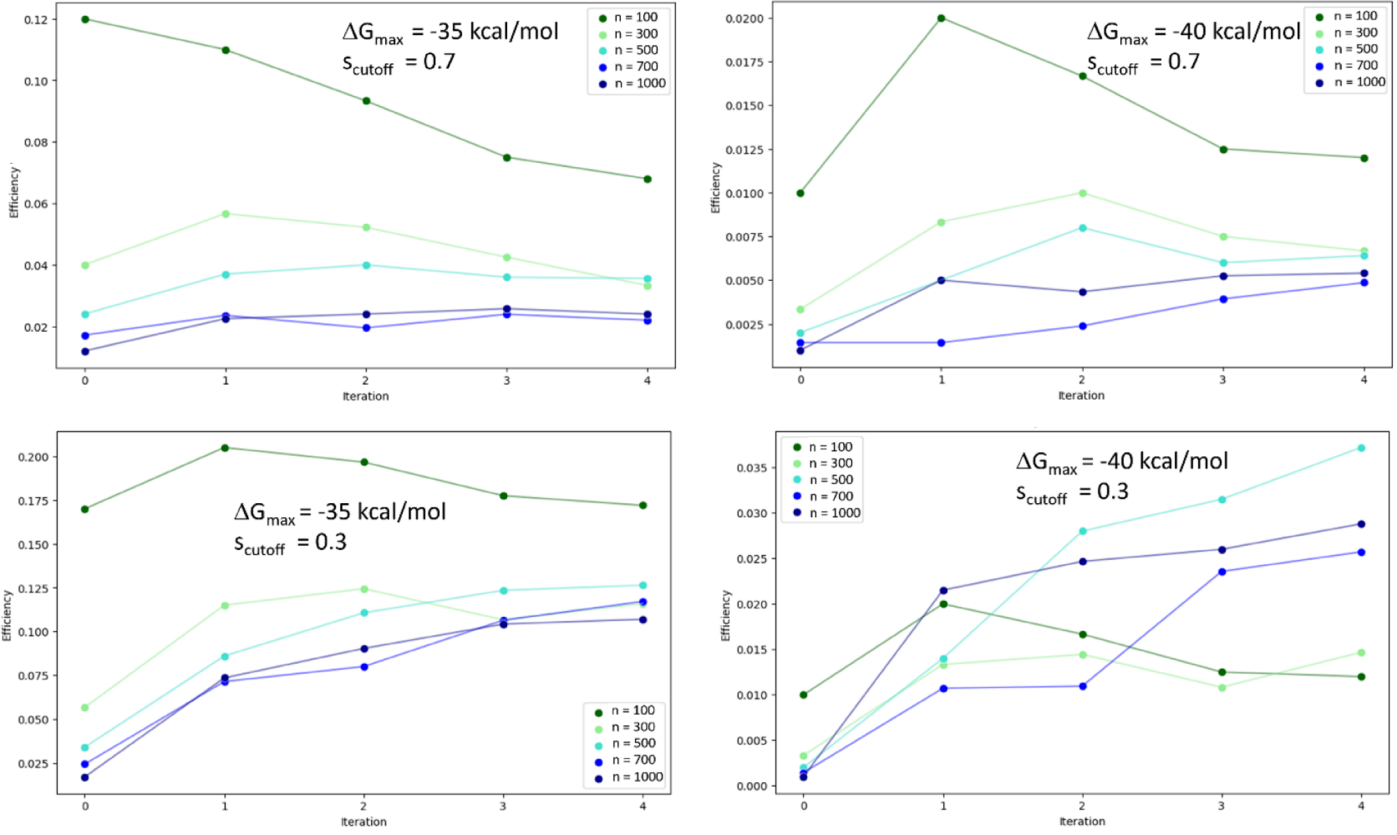
Efficiency of GAL for the TNKS2 systems using different training batch sizes, color coded as specified in the plot legends, after each iteration step. Efficiency is defined as the number of structural clusters found per oracle call of all cumulatively generated structures at a given iteration step. Only ligands with Δ*G*_b_ < Δ*G*_max_ were considered. Clustering was carried out using the Butina algorithm with a Tanimoto similarity cutoff given in respective plots.

The results for the computational efficiency η of GAL for TNKS2 are shown in Figure 15 for different training batch sizes, and two different values for Δ*G*_max_ and *s*_cutoff_. A value of Δ*G*_max_ = −35 kcal/mol describes approximately the maximum of the Δ*G*_b_ distributions obtained after GAL, whereas a value of −40 kcal/mol was chosen as a stricter criterion for selecting suitable compounds.

According to these results we observe improving efficiency within increasing iteration steps for larger batch sizes of *n* = 500 and larger, whereas for *n* = 100 efficiency remained the same or decreased. This finding is in line with the quality of the surrogate models, as shown in Figure 9. The more precise surrogate models for *n* ≥ 500 are more capable in identifying suitable new compounds, thereby increasing efficiency during GAL, however, at the cost of more numerous oracle calls. It turns out that smaller batch sizes were more efficient in most cases, especially the smallest batch size *n* = 100. Only in the case of Δ*G*_max_ = −40 kcal/mol and *s*_cutoff_ = 0.3 was a batch size of *n* = 500 found to be most efficient, followed by *n* = 1000 and *n* = 700. As explained in Section 3.2.5, these parameters define an η that is more suitable in a situation that prioritizes finding large binding affinities over chemical space exploration.

These findings indicate that a more explorative GAL would benefit from smaller training batch sizes, whereas exploitation would perform more efficiently with a large batch size. This finding is somewhat different from the situation in 3CL^pro^ where the smaller training batch size always led to a more efficient GAL. The likely cause for this difference is that for 3CL^pro^ the precision of the surrogate model remained limited in all cases compared to that for the TNKS2 case. It appears that, for a large training batch size to improve GAL efficiency, a sufficiently precise surrogate model is required. Overall, our findings indicate that in most scenarios a small batch size is more efficient and a safer choice when the quality of the surrogate model is *a priori* unknown.

## 4 Discussion

We have shown in this article that AL can be an effective method to improve compound optimization for a particular target. It is important to point out how the term “active learning” is being used as we follow here the convention which has been adopted in at least part of the community.^12−16,18−20^ However, we have also pointed out in the Introduction that we are not principally interested in creating a surrogate model for the purpose of optimally finding new labels i.e., binding affinities. In fact, in our application of AL the models (the surrogate model and the RL agent which is effectively a secondary surrogate model) are merely artifacts and the primary focus is in producing a sufficient number of high-scoring molecules as estimated by ESMACS. We are not truly trying to find the extremum of a function in the strict meaning of “optimization”. Figure S1 shows when high-scoring molecules are found over the course of an AL run, and we see that high-scoring molecules can be found very early in any RL cycle and also in any AL step. This is important in budget-constraint situations where only a limited amount of time or money is available. As we show, GAL is perfectly capable of finding quality compounds within such regimes. We note that RL is highly stochastic in nature and varying hyperparameters may influence chemical space exploration. This, however, means that replicate runs can lead to more diversity in generated molecules. Running multiple replicates may be impracticable though due to computational cost and budget constraints. Drug discovery today is still very much a “numbers-game”, meaning that a sufficient number of suggestions need to be generated (ideation) which are then separately assessed by medicinal chemists. We also stress that we have here used only a very limited number of scoring components which are nowhere near sufficient to assess the quality of a compound as a viable drug candidate. The current setup leaves out developability of a molecule i.e., synthesizability, PK/PD, ADMET, IP, formulation, physicochemical properties, and so on. In this sense, the current work is a proof-of-concept to show-case GAL as an effective and resource-saving tool for molecule optimization. Therefore, in future it will be necessary to strive for a more realistic target profile. The scoring components employed in this work are binding free energy prediction (the main oracle) and two scoring components (QED, chemical alerts) which are rather minimalistic models to restrain the chemistry generated. We have completely left out e.g., synthesizability which, however, is a major design goal in practical molecular design for obvious reasons. We also add that some of the generated structures, see e.g., Figure 5, may not be chemically stable and thus require careful postprocessing or need more sophisticated synthesizability assessments as discussed here. Thus, unsurprisingly, the chemical space explored with GAL cannot principally be expected to resemble the one from actually synthesized and experimentally confirmed binders. For the same reason, generated high-scoring molecules may not exhibit much similarity with previously reported binders which, however, may be beneficial to inform new molecule designs based on the newly found chemical space. In future, we plan to use in-house solutions like AiZynthFinder^76^ and others to address synthesizability and biology.

We also demonstrate the need for combining a ligand-based generative model with physics-based methods. It has been shown that structure-based methods significantly improve outcomes of generative models^77−79^ and it has been shown how physics-based MD descriptors are needed especially in low data regimes.^80^ Docking itself typically correlates rather poorly with experiment or with more accurate MD-based methods and we also observe this in the present study (see Figure S13). Hence, docking is often employed for enrichment and prefiltering as scoring functions tend to be rather inaccurate.^81,82^ Here, we use the physics-based method ESMACS to inform an RL agent via a surrogate model and demonstrate how this can drive our GAL workflow to efficiently generate better binders. Our work also shows that there are many opportunities to refine and improve the GAL workflow. We have built a fixed, static surrogate model via a deep learning model with set hyper-parameters and geometry. In principle, we would have to search all of model space to find models which are compatible with predicting binding free energies in accord with physics-based binding affinity prediction. We note here that this model is entirely ligand based and does not take protein structure or ligand-protein interactions into account. Earlier attempts to incorporate some structure-based information into AL has only shown limited utility.^13^ Previous RBFE studies have made use of automatic QSAR model building^15,17^ and we could make use of our own QSARtuna software.^38^ However, building a model with ChemProp is a rather time-consuming undertaking especially when making use of multifold cross-validation and ensembles. Depending on data and data size, the search for the best model may require the change of the model building algorithm during AL, e.g., we have used in this study a random tree model for the relatively small data set size of 27 ligands in the case of TNKS2 while for the actual AL runs we opted to seed with a rather large number of compounds where neural network models are much better suited.^32^ We have also created the surrogate model from “scratch” in every AL step i.e., starting anew from the accumulated compound data set where weights and biases were seeded from the previous AL step’s model. But we could make also use of layer-freezing techniques^32^ to accelerate surrogate model training. Likewise, the RL agent is being retrained in every AL step; we could check to see if the final or late-stage agent from the previous AL step could serve as a suitable starting point in the next GAL cycle. This would need to be gauged against concerns about model plasticity^83−85^ where covariate shift, which here comes in the form of changing chemical diversity, is one cause of concern. While AL is not continual learning this may still be an issue for even a limited number of steps of agent progression over time.

In the present work we have carried out a virtual screening or virtual hit finding exercise. The classical REINVENT prior enables de novo design which means that molecule generation is not limited by structural restraints but only by restraints stemming from the scoring components. In this sense we are carrying out inverse design, that is we create new molecules by describing the desired property space. For actual lead finding, in particular with AL and free energy simulation, it is typical to develop new chemistry around a common core or a limited number of cores which can be connected via a RBFE mapping network. To facilitate this with REINVENT we could make use of the Mol2Mol model^86^ which restrains molecule generation based on a similarity relationship of molecule pairs, e.g., using Tanimoto similarity or matched molecular pairs. This would enable us to modify a given scaffold itself depending on the tightness of the similarity criterion. If a scaffold constraint is desired our Libinvent model^87^ can be used and therefore generation of new molecules would occur around a fixed, preset core. Currently, REINVENT does not support fragment-based approaches directly i.e., our Linkinvent model^88^ only supports two “warheads” (fragments) which can then be linked with a single fragment. This could be extended to allow for multiple fragments. For example, the 3CL^pro^ target has a rather large, branched” binding site (see Figure 7) and so multiple known fragments in different binding site locations, as e.g., found in this study, could be joined together.

ESMACS is an ensemble MD method which means that multiple independent MD simulations of exactly the same molecular system are run to reinforce the statistical robustness of the simulation and its results. In the same vein, we can think about running REINVENT in an ensemble fashion. Similar to MD, the RL method is highly stochastic in nature and therefore it is advisible to carry out multiple RL runs too. There are various options available to do so. We could start multiple REINVENT runs from a single ChemProp model. We could also make use of multiple ChemProp models generated with cross-validation as several folds and also several ensembles within each fold can be made available for that^32^ as we have done here. It should be noted, however, that querying multiple such models will increase inference time; in fact, the scaling is O(N) with N being the number of models. Here, the time cost for a single inference step is about 1 s and 300–500 RL steps are carried out which would require around a day with 25 models (based on the hardware available in this study). It would therefore be more computationally efficient to infer from individual models in parallel and compute the statistics post hoc. In this way we could also make use of this as uncertainty quantification (UQ). It has been shown in an RBFE study,^14^ however, that including their uncertainty measure in the acquisition function only had a weak influence on AL performance.^14^ Alternatively, multiple independent surrogate models could be built, by varying geometries of a given network or make use of entirely different model algorithm as discussed above (resulting possibly in models of different quality/fidelity), or in a multifidelity fashion,^6,22,23^ where data of heterogeneous quality is used to create a single surrogate model.

The multifidelity approach has been shown to be particularly effective when a pretrained surrogate model is trained on data that is abundantly or easily available (docking, BindingDB data) and expensive high-fidelity data (ABFE) is added to the surrogate in small volumes.^23^ The authors note, however, that this approach could be outperformed by a surrogate built on high-fidelity data only. The surrogate model in this work is based on 10 000–13 000 data points. It is of note that their generative model^89^ appears to have been trained on the MOSES benchmark data set of compounds^90^ which is a subset of the lead-like (“rule of 3.5”) ZINC 12 Clean Lead data set.^91^ REINVENT^28^ priors are not limited to lead-like compounds. Some molecules generated in this work exhibit molecular masses larger than 500 Da. We also note that their generative model requires a QED weight twice as high as the ABFE component which indicates that the model struggles to produce high-quality drug-like compounds and may also reduce sample efficiency. Our weights are in the ratio 3:1 for the ABFE component to the QED component.

## 5 Conclusions

An AL protocol for the de novo design of protein ligands was introduced by combining generative molecular AI performed by REINVENT with physics-based ABFE scoring of ligands using ensemble MD simulations and MMPBSA. The latter uses a coarse-grained version of the ESMACS simulation protocol. The protocol, called GAL (generative active learning), was applied to two test target proteins, 3CL^pro^ and TNKS2. In both cases, compounds with large binding affinities were generated that were structurally diverse and with substantial variations in their scaffolds. At the same time, these structures were found to be very dissimilar to the structures that were initially used to train the surrogate model of our GAL workflow and different regions in chemical space were efficiently explored. We have found many compounds whose binding affinities exceed those of structures generated by extensive surrogate docking models in the case of 3CL^pro^ and those ligands that have been experimentally confirmed to exhibit strong binding affinities for TNKS2.

The narrowly confined binding pocket of TNKS2 led to a well-defined binding mode of ligands that translated into higher precision of GAL surrogate models for binding affinities, which in turn enabled finding structures with optimized binding affinities in fewer GAL iterations. In contrast, 3CL^pro^ is characterized by a larger open binding pocket that allows variations in binding modes and thereby resulted in surrogate models with reduced precision for which more GAL iterations were required. However, GAL performed successfully in both cases: only 3–4 iteration steps were required in the case of TNKS2 and 5–7 for 3CL^pro^, for generated structures to converge in chemical space. This convergence of GAL could be described as a transition from exploration of chemical space in the first iteration steps to a more exploitative regime, i.e., refinement of the structures discovered during the final iteration steps. This transition was not explicitly controlled, and all GAL steps were carried out using the same automated protocol, including the acquisition function.

Different GAL training batch sizes were tested, and qualitatively similar results were obtained. However, the larger batch sizes led in the case of TNKS2 to more precise surrogate models, accelerating convergence of GAL at the additional computational cost of more oracle calls. When comparing computational efficiency of different training batch sizes, smaller batch sizes were found to be more efficient in most cases. Considering that GAL performed successfully for all tested batch sizes, a suitable training batch size should be selected according to computational efficiency, which favored batch sizes 100–250 in this work.

Overall, even though there is clearly the potential for making further improvements, our REINVENT–ESMACS GAL protocol in its current form has been applied successfully as a generator of new compound ideas to two very different target proteins. We have demonstrated that this protocol, when integrated into a larger workflow to assess other important compound properties and to refine short-listed compounds further, is capable of *de novo* drug design to shorten the design-make-test-analysis cycle in drug discovery campaigns. It shows the immense potential of a combined AL and physics-based approach when employed reliably using ensemble simulations to control uncertainty, which is highly effective on a large supercomputer.

## Supporting Information Available

Additional graphs and material presenting results in more detail and graphs supporting discussion points in the main text, including input configuration for the generative AI software REINVENT (PDF)

## Abbreviations

3CL^pro^: 3C-like protease
TNKS2: Tankyrase-2
AL: active learning
GAL: generative active learning

## References

[ref1] Schlander M.; Hernandez-Villafuerte K.; Cheng C. Y.; Mestre-Ferrandiz J.; Baumann M. How Much Does It Cost to Research and Develop a New Drug? A Systematic Review and Assessment. PharmacoEconomics 2021, 39, 1243 10.1007/s40273-021-01065-y. 34368939 PMC8516790

[ref2] Wouters O. J.; McKee M.; Luyten J. Estimated Research and Development Investment Needed to Bring a New Medicine to Market, 2009–2018. JAMA 2020, 323, 844 10.1001/jama.2020.1166. 32125404 PMC7054832

[ref3] Wesolowski S. S.; Brown D. G. The Strategies and Politics of Successful Design, Make, Test, and Analyze (DMTA) Cycles inLead Generation. In Lead Generation; Wiley, 2016. 10.1002/9783527677047.ch17.

[ref4] Schneider P.; Walters W. P.; Plowright A. T.; Sieroka N.; Listgarten J.; Goodnow R. A.; Fisher J.; Jansen J. M.; Duca J. S.; Rush T. S.; Zentgraf M.; Hill J. E.; Krutoholow E.; Kohler M.; Blaney J.; Funatsu K.; Luebkemann C.; Schneider G. Rethinking Drug Design in the Artificial Intelligence Era. Nat. Rev. Drug Discovery 2020, 19, 353 10.1038/s41573-019-0050-3. 31801986

[ref5] Garnett R. Bayesian Optimization; Cambridge University Press, 2023.

[ref6] Di Fiore F.; Nardelli M.; Mainini L. Active Learning and Bayesian Optimization: A Unified Perspective to Learn with a Goal. Arch. Comput. Methods Eng. 2024, 31, 2985 10.1007/s11831-024-10064-z.

[ref7] Sugiyama M.; Nakajima S. Pool-Based Active Learning in Approximate Linear Regression. Mach. Learn. 2009, 75 (3), 249 10.1007/s10994-009-5100-3.

[ref8] Gentile F.; Agrawal V.; Hsing M.; Ton A. T.; Ban F.; Norinder U.; Gleave M. E.; Cherkasov A. Deep Docking: A Deep Learning Platform for Augmentation of Structure Based Drug Discovery. ACS Cent. Sci. 2020, 6 (6), 939 10.1021/acscentsci.0c00229. 32607441 PMC7318080

[ref9] Graff D. E.; Shakhnovich E. I.; Coley C. W. Accelerating High-Throughput Virtual Screening through Molecular Pool-Based Active Learning. Chem. Sci. 2021, 12 (22), 7866 10.1039/D0SC06805E. 34168840 PMC8188596

[ref10] Marin E.; Kovaleva M.; Kadukova M.; Mustafin K.; Khorn P.; Rogachev A.; Mishin A.; Guskov A.; Borshchevskiy V. Regression-Based Active Learning for Accessible Acceleration of Ultra-Large Library Docking. J. Chem. Inf. Model. 2024, 64, 2612 10.1021/acs.jcim.3c01661. 38157481 PMC11005039

[ref11] Bellmann L.; Penner P.; Gastreich M.; Rarey M. Comparison of Combinatorial Fragment Spaces and Its Application to Ultralarge Make-on-Demand Compound Catalogs. J. Chem. Inf. Model. 2022, 62 (3), 553 10.1021/acs.jcim.1c01378. 35050621

[ref12] Konze K. D.; Bos P. H.; Dahlgren M. K.; Leswing K.; Tubert-Brohman I.; Bortolato A.; Robbason B.; Abel R.; Bhat S. Reaction-Based Enumeration, Active Learning, and Free Energy Calculations to Rapidly Explore Synthetically Tractable Chemical Space and Optimize Potency of Cyclin-Dependent Kinase 2 Inhibitors. J. Chem. Inf. Model. 2019, 59 (9), 3782 10.1021/acs.jcim.9b00367. 31404495

[ref13] Khalak Y.; Tresadern G.; Hahn D. F.; De Groot B. L.; Gapsys V. Chemical Space Exploration with Active Learning and Alchemical Free Energies. J. Chem. Theory Comput. 2022, 18 (10), 6259 10.1021/acs.jctc.2c00752. 36148968 PMC9558370

[ref14] Thompson J.; Walters W. P.; Feng J. A.; Pabon N. A.; Xu H.; Maser M.; Goldman B. B.; Moustakas D.; Schmidt M.; York F. Optimizing Active Learning for Free Energy Calculations. Artif. Intell. Life Sci. 2022, 2, 100050 10.1016/j.ailsci.2022.100050.

[ref15] Gusev F.; Gutkin E.; Kurnikova M. G.; Isayev O. Active Learning Guided Drug Design Lead Optimization Based on Relative Binding Free Energy Modeling. J. Chem. Inf. Model. 2023, 63 (2), 583 10.1021/acs.jcim.2c01052. 36599125

[ref16] Crivelli-Decker J. E.; Beckwith Z.; Tom G.; Le L.; Khuttan S.; Salomon-Ferrer R.; Beall J.; Gómez-Bombarelli R.; Bortolato A. Machine Learning Guided AQFEP: A Fast & Efficient Absolute Free Energy Perturbation Solution for Virtual Screening. ChemRxiv 2023, 10.26434/chemrxiv-2023-z3t3b. PMC11360131 39146234

[ref17] de Oliveira C.; Leswing K.; Feng S.; Kanters R.; Abel R.; Bhat S. FEP Protocol Builder: Optimization of Free Energy Perturbation Protocols Using Active Learning. J. Chem. Inf. Model. 2023, 63 (17), 5592–5603. 10.1021/acs.jcim.3c00681. 37594480

[ref18] Knight J. L.; Leswing K.; Bos P. H.; Wang L. Impacting Drug Discovery Projects with Large-Scale Enumerations, Machine Learning Strategies, and Free-Energy Predictions. ACS Symp. Ser. 2021, 1397, 205 10.1021/bk-2021-1397.ch008.

[ref19] Mohr B.; Shmilovich K.; Kleinwächter I. S.; Schneider D.; Ferguson A. L.; Bereau T. Data-Driven Discovery of Cardiolipin-Selective Small Molecules by Computational Active Learning. Chem. Sci. 2022, 13, 4498 10.1039/D2SC00116K. 35656132 PMC9019913

[ref20] Gorantla R.; Kubincová A.; Suutari B.; Cossins B. P.; Mey A. S. J. S. Benchmarking Active Learning Protocols for Ligand-Binding Affinity Prediction. J. Chem. Inf. Model. 2024, 64 (6), 1955–1965. 10.1021/acs.jcim.4c00220. 38446131 PMC10966646

[ref21] Filella-Merce I.; Molina A.; Orzechowski M.; Díaz L.; Zhu Y. M.; Mor J. V.; Malo L.; Yekkirala A. S.; Ray S.; Guallar V. Optimizing Drug Design by Merging Generative AI With Active Learning Frameworks arXiv 2023 10.48550/arXiv.2305.06334. PMC12334747 40781156

[ref22] Hernandez-Garcia A.; Saxena N.; Jain M.; Liu C.-H.; Bengio Y. Multi-Fidelity Active Learning with GFlowNets. arXiv 2023, 10.48550/arXiv.2306.11715.

[ref23] Eckmann P.; Wu D.; Heinzelmann G.; Gilson M. K.; Yu R. MFBind: A Multi-Fidelity Approach for Evaluating Drug Compounds in Practical Generative Modeling arXiv 2024 10.48550/arXiv.2402.10387.

[ref24] Liu T.; Lin Y.; Wen X.; Jorissen R. N.; Gilson M. K. BindingDB: A Web-Accessible Database of Experimentally Determined Protein-Ligand Binding Affinities. Nucleic Acids Res. 2007, 35 (SUPPL. 1), D198–D201. 10.1093/nar/gkl999. 17145705 PMC1751547

[ref25] Heinzelmann G.; Gilson M. K. Automation of Absolute Protein-Ligand Binding Free Energy Calculations for Docking Refinement and Compound Evaluation. Sci. Rep. 2021, 11 (1), 1116 10.1038/s41598-020-80769-1. 33441879 PMC7806944

[ref26] Wu D. Pool-Based Sequential Active Learning for Regression. IEEE Trans. Neural Netw. Learn. Syst. 2019, 30 (5), 1348 10.1109/TNNLS.2018.2868649. 30281482

[ref27] Kearns M.; Rubinfeld R.; Mansour Y.; Schapire R. E.; Ron D.; Univemity H. On the Learnability of Discrete Distributions. Proc. Annu. ACM Symp. Theory Comput. 1994, 273 10.1145/195058.195155.

[ref28] Loeffler H. H.; He J.; Tibo A.; Janet J. P.; Voronov A.; Mervin L. H.; Engkvist O. Reinvent 4: Modern AI–Driven Generative Molecule Design. J. Cheminform. 2024, 16 (1), 20 10.1186/s13321-024-00812-5. 38383444 PMC10882833

[ref29] Arús-Pous J.; Blaschke T.; Ulander S.; Reymond J. L.; Chen H.; Engkvist O. Exploring the GDB-13 Chemical Space Using Deep Generative Models. J. Cheminform. 2019, 11 (1), 20 10.1186/s13321-019-0341-z. 30868314 PMC6419837

[ref30] Stokes J. M.; Yang K.; Swanson K.; Jin W.; Cubillos-Ruiz A.; Donghia N. M.; MacNair C. R.; French S.; Carfrae L. A.; Bloom-Ackerman Z.; Tran V. M.; Chiappino-Pepe A.; Badran A. H.; Andrews I. W.; Chory E. J.; Church G. M.; Brown E. D.; Jaakkola T. S.; Barzilay R.; Collins J. J. A Deep Learning Approach to Antibiotic Discovery. Cell 2020, 180 (4), 688–702.e13. 10.1016/j.cell.2020.01.021. 32084340 PMC8349178

[ref31] Heid E.; Green W. H. Machine Learning of Reaction Properties via Learned Representations of the Condensed Graph of Reaction. J. Chem. Inf. Model. 2022, 62, 2101 10.1021/acs.jcim.1c00975. 34734699 PMC9092344

[ref32] Heid E.; Greenman K. P.; Chung Y.; Li S.-C.; Graff D. E.; Vermeire F. H.; Wu H.; Green W. H.; McGill C. J. Chemprop: A Machine Learning Package for Chemical Property Prediction. J. Chem. Inf. Model. 2024, 64 (1), 9–17. 10.1021/acs.jcim.3c01250. 38147829 PMC10777403

[ref33] Yang K.; Swanson K.; Jin W.; Coley C.; Eiden P.; Gao H.; Guzman-Perez A.; Hopper T.; Kelley B.; Mathea M.; Palmer A.; Settels V.; Jaakkola T.; Jensen K.; Barzilay R. Analyzing Learned Molecular Representations for Property Prediction. J. Chem. Inf. Model. 2019, 59 (8), 3370 10.1021/acs.jcim.9b00237. 31361484 PMC6727618

[ref34] Clyde A.; Liu X.; Brettin T.; Yoo H.; Partin A.; Babuji Y.; Blaiszik B.; Mohd-Yusof J.; Merzky A.; Turilli M.; Jha S.; Ramanathan A.; Stevens R. AI-Accelerated Protein-Ligand Docking for SARS-CoV-2 Is 100-Fold Faster with No Significant Change in Detection. Sci. Rep. 2023, 13 (1), 2105 10.1038/s41598-023-28785-9. 36747041 PMC9901402

[ref35] Clyde A.; Galanie S.; Kneller D. W.; Ma H.; Babuji Y.; Blaiszik B.; Brace A.; Brettin T.; Chard K.; Chard R.; Coates L.; Foster I.; Hauner D.; Kertesz V.; Kumar N.; Lee H.; Li Z.; Merzky A.; Schmidt J. G.; Tan L.; Titov M.; Trifan A.; Turilli M.; Van Dam H.; Chennubhotla S. C.; Jha S.; Kovalevsky A.; Ramanathan A.; Head M. S.; Stevens R. High-Throughput Virtual Screening and Validation of a SARS-CoV-2 Main Protease Noncovalent Inhibitor. J. Chem. Inf. Model. 2022, 62 (1), 116 10.1021/acs.jcim.1c00851. 34793155

[ref36] Stone M. Cross-Validatory Choice and Assessment of Statistical Predictions. J. R. Stat. Soc. 1974, 36 (2), 111 10.1111/j.2517-6161.1974.tb00994.x.

[ref37] Schindler C. E. M.; Baumann H.; Blum A.; Böse D.; Buchstaller H. P.; Burgdorf L.; Cappel D.; Chekler E.; Czodrowski P.; Dorsch D.; Eguida M. K. I.; Follows B.; Fuchß T.; Grädler U.; Gunera J.; Johnson T.; Lebrun C. J.; Karra S.; Klein M.; Knehans T.; Koetzner L.; Krier M.; Leiendecker M.; Leuthner B.; Li L.; Mochalkin I.; Musil D.; Neagu C.; Rippmann F.; Schiemann K.; Schulz R.; Steinbrecher T.; Tanzer E. M.; Lopez A. U.; Follis A. V.; Wegener A.; Kuhn D. Large-Scale Assessment of Binding Free Energy Calculations in Active Drug Discovery Projects. J. Chem. Inf. Model. 2020, 60 (11), 5457–5474. 10.1021/acs.jcim.0c00900. 32813975

[ref38] Mervin L.; Voronov A.; Kabeshov M.; Engkvist O. QSARtuna: An Automated QSAR Modeling Platform for Molecular Property Prediction in Drug Design. J. Chem. Inf. Model. 2024, 64 (14), 5365–5374. 10.1021/acs.jcim.4c00457. 38950185

[ref39] Richard Bickerton G.; Paolini G. V.; Besnard J.; Muresan S.; Hopkins A. L. Quantifying the Chemical Beauty of Drugs. Nat. Chem. 2012, 4 (2), 90–98. 10.1038/nchem.1243. 22270643 PMC3524573

[ref40] Olivecrona M.; Blaschke T.; Engkvist O.; Chen H. Molecular De-Novo Design through Deep Reinforcement Learning. J. Cheminform. 2017, 9 (1), 48 10.1186/s13321-017-0235-x. 29086083 PMC5583141

[ref41] Blaschke T.; Arús-Pous J.; Chen H.; Margreitter C.; Tyrchan C.; Engkvist O.; Papadopoulos K.; Patronov A. REINVENT 2.0: An AI Tool for de Novo Drug Design. J. Chem. Inf. Model. 2020, 60 (12), 5918 10.1021/acs.jcim.0c00915. 33118816

[ref42] Fialková V.; Zhao J.; Papadopoulos K.; Engkvist O.; Bjerrum E. J.; Kogej T.; Patronov A. LibINVENT Reaction-Based Generative Scaffold Decoration for in Silico Library Design. J. Chem. Inf. Model. 2022, 62, 2046 10.1021/acs.jcim.1c00469. 34460269

[ref43] Bemis G. W.; Murcko M. A. The Properties of Known Drugs. 1. Molecular Frameworks. J. Med. Chem. 1996, 39 (15), 2887 10.1021/jm9602928. 8709122

[ref44] RDKit 2022.09.5: Open-Source Cheminformatics; Zenodo, 2022. 10.5281/zenodo.7671152.

[ref45] Blaschke T.; Engkvist O.; Bajorath J.; Chen H. Memory-Assisted Reinforcement Learning for Diverse Molecular de Novo Design. J. Cheminform. 2020, 12 (1), 68 10.1186/s13321-020-00473-0. 33292554 PMC7654024

[ref46] Wan S.; Bhati A. P.; Zasada S. J.; Coveney P. V. Rapid, accurate, precise and reproducible ligand–protein binding free energy prediction. Interface Focus 2020, 10, 20200007 10.1098/rsfs.2020.0007. 33178418 PMC7653346

[ref47] Wan S.; Knapp B.; Wright D. W.; Deane C. M.; Coveney P. V. Rapid Precise, and Reproducible Prediction of Peptide-MHC Binding Affinities from Molecular Dynamics That Correlate Well with Experiment. J. Chem. Theory Comput. 2015, 11 (7), 3346–3356. 10.1021/acs.jctc.5b00179. 26575768

[ref48] Homeyer N.; Gohlke H. Free Energy Calculations by the Molecular Mechanics Poisson-Boltzmann Surface Area Method. Mol. Inform. 2012, 31 (2), 114–122. 10.1002/minf.201100135. 27476956

[ref49] OpenEye, Cadence Molecular Sciences, Inc. NM, Santa Fe. https://www.eyesopen.com/. (accessed 2024 August 05).

[ref50] Hawkins P. C. D.; Skillman A. G.; Warren G. L.; Ellingson B. A.; Stahl M. T. Conformer Generation with OMEGA: Algorithm and Validation Using High Quality Structures from the Protein Databank and Cambridge Structural Database. J. Chem. Inf. Model. 2010, 50 (4), 572 10.1021/ci100031x. 20235588 PMC2859685

[ref51] McGann M. FRED Pose Prediction and Virtual Screening Accuracy. J. Chem. Inf. Model. 2011, 51 (3), 578 10.1021/ci100436p. 21323318

[ref52] McGann M. FRED and HYBRID Docking Performance on Standardized Datasets. J. Comput.-Aided Mol. Des. 2012, 26 (8), 897–906. 10.1007/s10822-012-9584-8. 22669221

[ref53] Sadiq S. K.; Wright D.; Watson S. J.; Zasada S. J.; Stoica I.; Coveney P. V. Automated Molecular Simulation Based Binding Affinity Calculator for Ligand-Bound HIV-1 Proteases Automated Molecular Simulation Based Binding Affinity Calculator for Ligand-Bound HIV-1 Proteases. Mol. Simul. 2008, 48 (Md), 1909–1919. 10.1021/ci8000937. 18710212

[ref54] Case D. A.; Aktulga H. M.; Belfon K.; Cerutti D. S.; Cisneros G. A.; Cruzeiro V. W. D.; Forouzesh N.; Giese T. J.; Götz A. W.; Gohlke H.; Izadi S.; Kasavajhala K.; Kaymak M. C.; King E.; Kurtzman T.; Lee T. S.; Li P.; Liu J.; Luchko T.; Luo R.; Manathunga M.; Machado M. R.; Nguyen H. M.; O’Hearn K. A.; Onufriev A. V.; Pan F.; Pantano S.; Qi R.; Rahnamoun A.; Risheh A.; Schott-Verdugo S.; Shajan A.; Swails J.; Wang J.; Wei H.; Wu X.; Wu Y.; Zhang S.; Zhao S.; Zhu Q.; Cheatham T. E.; Roe D. R.; Roitberg A.; Simmerling C.; York D. M.; Nagan M. C.; Merz K. M. AmberTools. J. Chem. Inf. Model. 2023, 63 (20), 6183–6191. 10.1021/acs.jcim.3c01153. 37805934 PMC10598796

[ref56] Wan S.; Bhati A. P.; Wade A. D.; Coveney P. V. Ensemble-Based Approaches Ensure Reliability and Reproducibility. J. Chem. Inf. Model. 2023, 63, 6959–6963. 10.1021/acs.jcim.3c01654. 37965695 PMC10685440

[ref57] Phillips J. C.; Hardy D. J.; Maia J. D. C.; Stone J. E.; Ribeiro J. V.; Bernardi R. C.; Buch R.; Fiorin G.; Hénin J.; Jiang W.; McGreevy R.; Melo M. C. R.; Radak B. K.; Skeel R. D.; Singharoy A.; Wang Y.; Roux B.; Aksimentiev A.; Luthey-Schulten Z.; Kalé L. V.; Schulten K.; Chipot C.; Tajkhorshid E. Scalable Molecular Dynamics on CPU and GPU Architectures with NAMD. J. Chem. Phys. 2020, 153 (4), 044130 10.1063/5.0014475. 32752662 PMC7395834

[ref58] Warmuth M. K.; Liao J.; Rätsch G.; Mathieson M.; Putta S.; Lemmen C. Active Learning with Support Vector Machines in the Drug Discovery Process. J. Chem. Inf. Comput. Sci. 2003, 43, 667 10.1021/ci025620t. 12653536

[ref59] McCloskey M.; Cohen N. J. Catastrophic Interference in Connectionist Networks: The Sequential Learning Problem. Psychol. Learn. Motiv. 1989, 24, 109 10.1016/S0079-7421(08)60536-8.

[ref60] McInnes L.; Healy J.; Saul N.; Großberger L. UMAP: Uniform Manifold Approximation and Projection. J. Open Source Softw. 2018, 3 (29), 861 10.21105/joss.00861.

[ref61] Bajusz D.; Rácz A.; Héberger K. Why Is Tanimoto Index an Appropriate Choice for Fingerprint-Based Similarity Calculations?. J. Cheminform. 2015, 7 (1), 20 10.1186/s13321-015-0069-3. 26052348 PMC4456712

[ref62] WHO chief declares end to COVID-19 as a global health emergency. https://news.un.org/en/story/2023/05/1136367. (accessed 2024 February 22).

[ref63] Kim M. K. Novel Insight into the Function of Tankyrase (Review). Oncol. Lett. 2018, 16, 6895 10.3892/ol.2018.9551. 30546421 PMC6256358

[ref64] Wang E.; Sun H.; Wang J.; Wang Z.; Liu H.; Zhang J. Z. H.; Hou T. End-Point Binding Free Energy Calculation with MM/PBSA and MM/GBSA: Strategies and Applications in Drug Design. Chem. Rev. 2019, 119, 9478 10.1021/acs.chemrev.9b00055. 31244000

[ref65] Wan S.; Bhati A. P.; Wright D. W.; Wade A. D.; Tresadern G.; van Vlijmen H.; Coveney P. V. The Performance of Ensemble-Based Free Energy Protocols in Computing Binding Affinities to ROS1 Kinase. Sci. Rep. 2022, 12 (1), 10433 10.1038/s41598-022-13319-6. 35729177 PMC9211793

[ref66] Wan S.; Bhati A. P.; Wright D. W.; Wall I. D.; Graves A. P.; Green D.; Coveney P. V. Ensemble Simulations and Experimental Free Energy Distributions: Evaluation and Characterization of Isoxazole Amides as SMYD3 Inhibitors. J. Chem. Inf. Model. 2022, 62, 2561 10.1021/acs.jcim.2c00255. 35508076 PMC9131449

[ref67] Wan S.; Potterton A.; Husseini F. S.; Wright D. W.; Heifetz A.; Malawski M.; Townsend-Nicholson A.; Coveney P. V. Hit-to-Lead and Lead Optimization Binding Free Energy Calculations for G Protein-Coupled Receptors: Free Energy Calculations for GPCRs. Interface Focus 2020, 10 (6), 20190128 10.1098/rsfs.2019.0128. 33178414 PMC7653344

[ref68] Wan S.; Bhati A. P.; Wade A. D.; Alfè D.; Coveney P. V. Thermodynamic and Structural Insights into the Repurposing of Drugs That Bind to SARS-CoV-2 Main Protease. Mol. Syst. Des. Eng. 2022, 7 (2), 123 10.1039/D1ME00124H. 35223088 PMC8820189

[ref69] Landrum G. A.; Riniker S. Combining IC50 or Ki Values from Different Sources Is a Source of Significant Noise. J. Chem. Inf. Model. 2024, 64 (5), 1560 10.1021/acs.jcim.4c00049. 38394344 PMC10934815

[ref70] Kalliokoski T.; Kramer C.; Vulpetti A.; Gedeck P.; Cavalli A. Comparability of Mixed IC50 Data – A Statistical Analysis. PLoS One 2013, 8 (4), e61007 10.1371/journal.pone.0061007. 23613770 PMC3628986

[ref71] Benhenda M. ChemGAN Challenge for Drug Discovery: Can AI Reproduce Natural Chemical Diversity?. arXiv 2017, 10.48550/arXiv.1708.08227.

[ref72] Morgan H. L. The Generation of a Unique Machine Description for Chemical Structures—A Technique Developed at Chemical Abstracts Service. J. Chem. Doc. 1965, 5 (2), 107 10.1021/c160017a018.

[ref73] Rogers D.; Hahn M. Extended-Connectivity Fingerprints. J. Chem. Inf. Model. 2010, 50 (5), 742 10.1021/ci100050t. 20426451

[ref74] Butina D. Unsupervised Data Base Clustering Based on Daylight’s Fingerprint and Tanimoto Similarity: A Fast and Automated Way to Cluster Small and Large Data Sets. J. Chem. Inf. Comput. Sci. 1999, 39 (4), 747 10.1021/ci9803381.

[ref75] Van Der Maaten L.; Hinton G. Visualizing Data Using T-SNE. J Mach. Learn. Res. 2008, 9, 2579–2605.

[ref76] Genheden S.; Thakkar A.; Chadimová V.; Reymond J. L.; Engkvist O.; Bjerrum E. AiZynthFinder: A Fast, Robust and Flexible Open-Source Software for Retrosynthetic Planning. J. Cheminform. 2020, 12 (1), 70 10.1186/s13321-020-00472-1. 33292482 PMC7672904

[ref77] Thomas M.; Bender A.; de Graaf C. Integrating Structure-Based Approaches in Generative Molecular Design. Curr. Opin. Struct. Biol. 2023, 79, 102559 10.1016/j.sbi.2023.102559. 36870277

[ref78] Papadopoulos K.; Giblin K. A.; Janet J. P.; Patronov A.; Engkvist O. De Novo Design with Deep Generative Models Based on 3D Similarity Scoring. Bioorg. Med. Chem. 2021, 44, 116308 10.1016/j.bmc.2021.116308. 34280849

[ref79] Sauer S.; Matter H.; Hessler G.; Grebner C. Optimizing Interactions to Protein Binding Sites by Integrating Docking-Scoring Strategies into Generative AI Methods. Front. Chem. 2022, 10, 101250 10.3389/fchem.2022.1012507. PMC9629386 36339033

[ref80] Chew A. K.; Sender M.; Kaplan Z.; Chandrasekaran A.; Chief Elk J.; Browning A. R.; Kwak H. S.; Halls M. D.; Afzal M. A. F. Advancing Material Property Prediction: Using Physics-Informed Machine Learning Models for Viscosity. J. Cheminform. 2024, 16 (1), 31 10.1186/s13321-024-00820-5. 38486289 PMC10938832

[ref81] Xu X.; Huang M.; Zou X. Docking-Based Inverse Virtual Screening: Methods, Applications, and Challenges. Biophys. Rep. 2018, 4 (1), 1 10.1007/s41048-017-0045-8. 29577065 PMC5860130

[ref82] Li J.; Fu A.; Zhang L. An Overview of Scoring Functions Used for Protein–Ligand Interactions in Molecular Docking. Interdiscip. Sci.-Comput. Life Sci. 2019, 11, 320–328. 10.1007/s12539-019-00327-w. 30877639

[ref83] Lyle C.; Zheng Z.; Khetarpal K.; van Hasselt H.; Pascanu R.; Martens J.; Dabney W. Disentangling the Causes of Plasticity Loss in Neural Networks. arXiv 2024, 10.48550/arXiv.2402.18762.

[ref84] Abbas Z.; Zhao R.; Modayil J.; White A.; Machado M. C. Loss of Plasticity in Continual Deep Reinforcement Learning. arXiv 2023, 10.48550/arXiv.2303.07507.

[ref85] Dohare S.; Sutton R. S.; Mahmood A. R. Continual Backprop: Stochastic Gradient Descent with Persistent Randomness. arXiv 2021, 10.48550/arXiv.2108.06325.

[ref86] He J.; Tibo A.; Janet J. P.; Nittinger E.; Tyrchan C.; Czechtizky W.; Ola E. Evaluation of Reinforcement Learning in Transformer-Based Molecular Design. J. Cheminf. 2024, 16, 95 10.1186/s13321-024-00887-0. PMC11312936 39118113

[ref87] Fialková V.; Zhao J.; Papadopoulos K.; Engkvist O.; Bjerrum E. J.; Kogej T.; Patronov A. LibINVENT: Reaction-Based Generative Scaffold Decoration for in Silico Library Design. J. Chem. Inf. Model. 2022, 62 (9), 2046–2063. 10.1021/acs.jcim.1c00469. 34460269

[ref88] Guo J.; Knuth F.; Margreitter C.; Janet J. P.; Papadopoulos K.; Engkvist O.; Patronov A. Link-INVENT: Generative Linker Design with Reinforcement Learning. Digital Discovery 2023, 2 (2), 392–408. 10.1039/D2DD00115B.

[ref89] Eckmann P.; Sun K.; Zhao B.; Feng M.; Gilson M. K.; Yu R. LIMO Latent Inceptionism for Targeted Molecule Generation. arXiv 2022, 10.48550/arXiv.2206.09010. PMC9527083 36193121

[ref90] Polykovskiy D.; Zhebrak A.; Sanchez-Lengeling B.; Golovanov S.; Tatanov O.; Belyaev S.; Kurbanov R.; Artamonov A.; Aladinskiy V.; Veselov M.; et al. Molecular Sets (MOSES): A Benchmarking Platform for Molecular Generation Models. Front. Pharmacol. 2020, 11, 565644 10.3389/fphar.2020.565644. 33390943 PMC7775580

[ref91] Irwin J. J.; Sterling T.; Mysinger M. M.; Bolstad E. S.; Coleman R. G. ZINC: A Free Tool to Discover Chemistry for Biology. J. Chem. Inf. Model. 2012, 52, 1757 10.1021/ci3001277. 22587354 PMC3402020

