# Supplementary material for: Optimal Molecular Design: Generative Active Learning Combining REINVENT with Precise Binding Free Energy Ranking Simulations
Source: J Chem Theory Comput. 2024 Sep 3;20(18):8308–28. doi: 10.1021/acs.jctc.4c00576 (PMC11428133; doi:10.1021/acs.jctc.4c00576)
Supplement: Supplementary file 1 — ct4c00576_si_001.pdf [file ct4c00576_si_001.pdf]

Supporting Information: Optimal Molecular Design: Generative Active Learning Combining REINVENT with Precise Binding Free Energy Ranking Simulations

Hannes H Loeffler[1]‡, Shunzhou Wan[2‡], Marco Klähn[1‡], Agastya P Bhati[2]*, Peter V Coveney[2,3,4]*

[1]Molecular AI, Discovery Sciences, R&D, AstraZeneca, 431 83 Mölndal, Sweden

[2]Centre for Computational Science, Department of Chemistry, University College London, London WC1H 0AJ, U. K.

[3]Advanced Research Computing Centre, University College London, London WC1H 0AJ, U.K.

,[4]Institute for Informatics, Faculty of Science, University of Amsterdam, 1098XH Amsterdam, The Netherlands.

Active Learning, Molecule Optimization, Generative AI, Absolute Binding Free Energies

When were high-scoring molecules generated and how many?

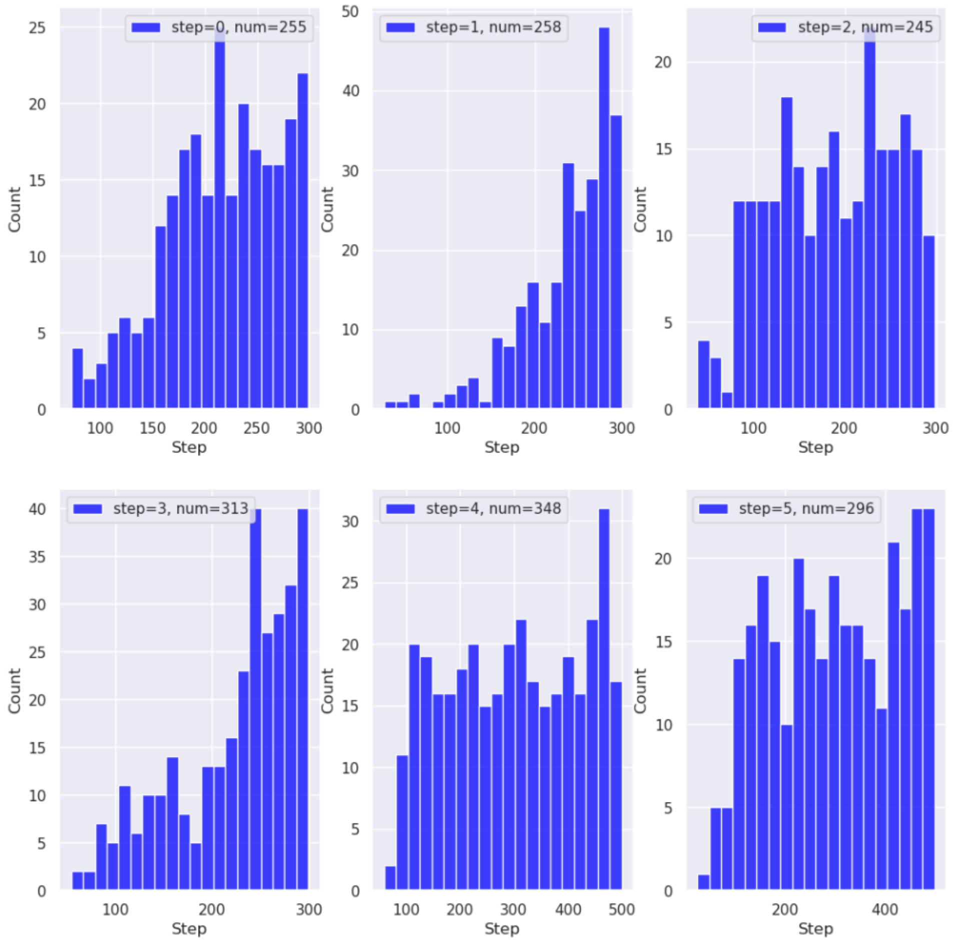

Figure S1. Histograms showing for each GAL step when high-scoring molecules (ΔG < -30 kcal/mol) were generated during reinforcement learning (RL). The number of total high-scoring molecules is shown in the legend. The run is for batch size=500 of 6 steps of GAL with 3CL^{pro}. Step numbers in each RL epoch vary as the protocol was adjusted in protocol development.

REINVENT configuration file

Scheme 1. TOML input configuration file for reinforcement learning (single stage staged learning).

```
# stage2.toml
run_type = "staged_learning"
use_cuda = true
tb_logdir = "tb_stage2"
json_out_config = "_stage2.json"
[parameters]
use_checkpoint = false
prior_file = "$PATH/reinvent/priors/reinvent.prior"
agent_file = "$PATH/reinvent/stage1.chkpt"
summary_csv_prefix = "stage2"
batch_size = 100
randomize_smiles = true
[learning_strategy]
type = "dap"
sigma = 128
rate = 0.0001
[diversity_filter]
type = "IdenticalMurckoScaffold"
bucket_size = 10
minscore = 0.7
minsimilarity = 0.5
[inception]
smiles_file = "exp27.smi"
memory_size = 50
sample_size = 10
[[stage]]
termination = "simple"
max_score = 1.0
max_steps = 500
chkpt_file = 'stage2.chkpt'
[stage.scoring]
type = "geometric_mean"
filename = "stage2_scoring.toml"
filetype = "TOML"


# stage2_scoring.toml
[[component]]
[component.custom_alerts]
[[component.custom_alerts.endpoint]]
name = "Alerts"
params.smarts = [
    "[*;r8]",
    "[*;r9]",
    "[*;r10]",
    "[*;r11]",
    "[*;r12]",
```

```
    "[*;r13]",
    "[*;r14]",
    "[*;r15]",
    "[*;r16]",
    "[*;r17]",
    "[#8][#8]",
    "[#6;+]",
    "[#16][#16]",
    "[#7;!n][S;!$(S(=O)=O)]",
    "[#7;!n][#7;!n]",
    "C#C",
    "C(=[O,S])[O,S]",
    "[#7;!n][C;!$(C(=[O,N])[N,O])][#16;!s]",
    "[#7;!n][C;!$(C(=[O,N])[N,O])][#7;!n]",
    "[#7;!n][C;!$(C(=[O,N])[N,O])][#8;!o]",
    "[#8;!o][C;!$(C(=[O,N])[N,O])][#16;!s]",
    "[#8;!o][C;!$(C(=[O,N])[N,O])][#8;!o]",
    "[#16;!s][C;!$(C(=[O,N])[N,O])][#16;!s]"
]
[[component]]
[component.QED]
[[component.QED.endpoint]]
name = "QED"
weight = 0.2
[[component]]
[component.NumAtomStereoCenters]
[[component.NumAtomStereoCenters.endpoint]]
name = "Stereo"
weight = 0.2
transform.type = "left_step"
transform.low = 0
[[component]]
[component.ChemProp]
[[component.ChemProp.endpoint]]
name = "ChemProp"
weight = 0.6
params.checkpoint_dir = "chemprop/fold_3/model_2/"
params.rdkit_2d_normalized = true
transform.type = "reverse_sigmoid"
transform.high = 0.0
transform.low = -50.0
transform.k = 0.4
```

Additional figures

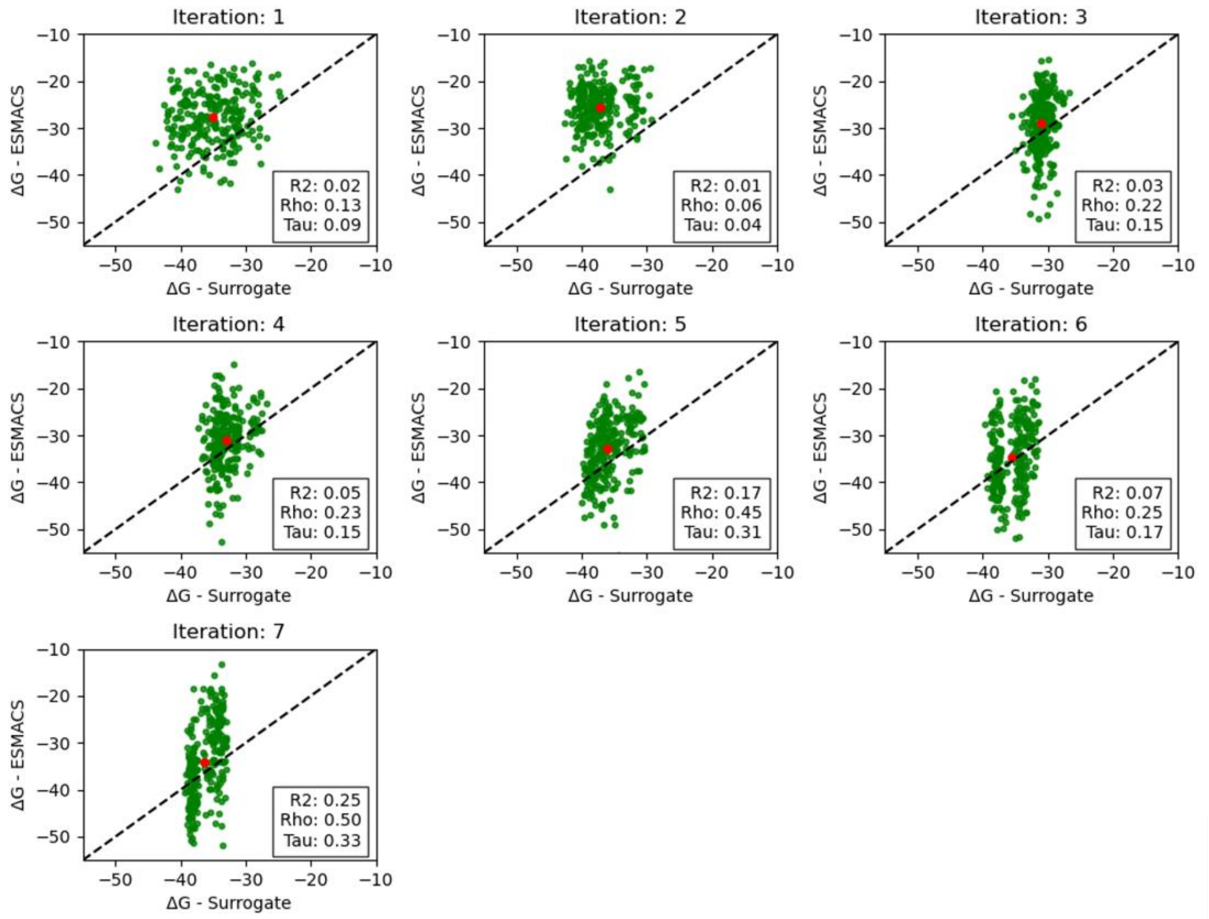

Figure S2. Comparison of surrogate model predictions of ΔG with calculated ESMACS values for training batch sizes of 250 molecules for each GAL iteration step for $3CL^{pro}$. R^2-coefficient as well as Spearman and Kendall rank correlation coefficients rho and tau are given in the insets of each plot. The average ΔG of all surrogate model predictions and ESMACS calculations within an iteration is shown as a red circle. All energies are given in units of kcal/mol.

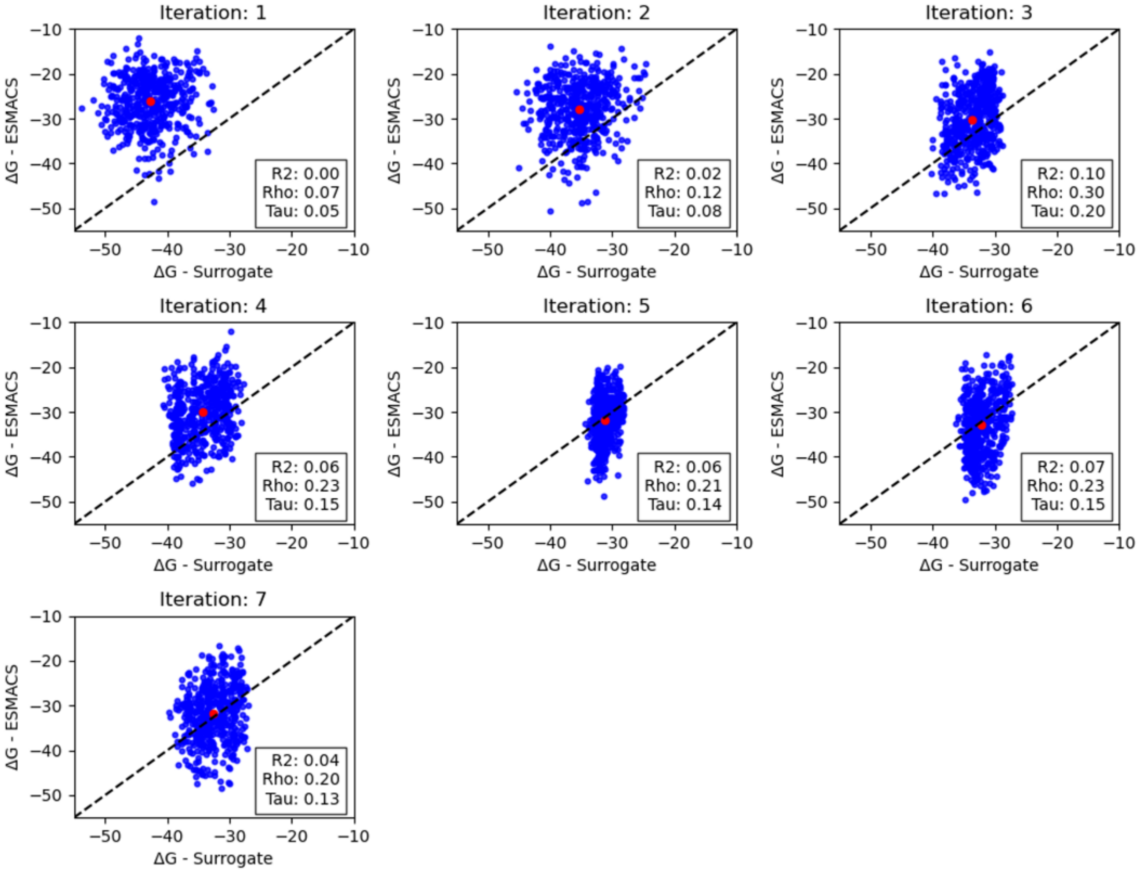

Figure S3. Comparison of surrogate model predictions of ΔG with calculated ESMACS values for training batch sizes of 500 molecules for each GAL iteration step for 3CL^{pro}. R^2-coefficient as well as Spearman and Kendall rank correlation coefficients rho and tau are given in the insets of each plot. The average ΔG of all surrogate model predictions and ESMACS calculations within an iteration is shown as a red circle. All energies are given in units of kcal/mol.

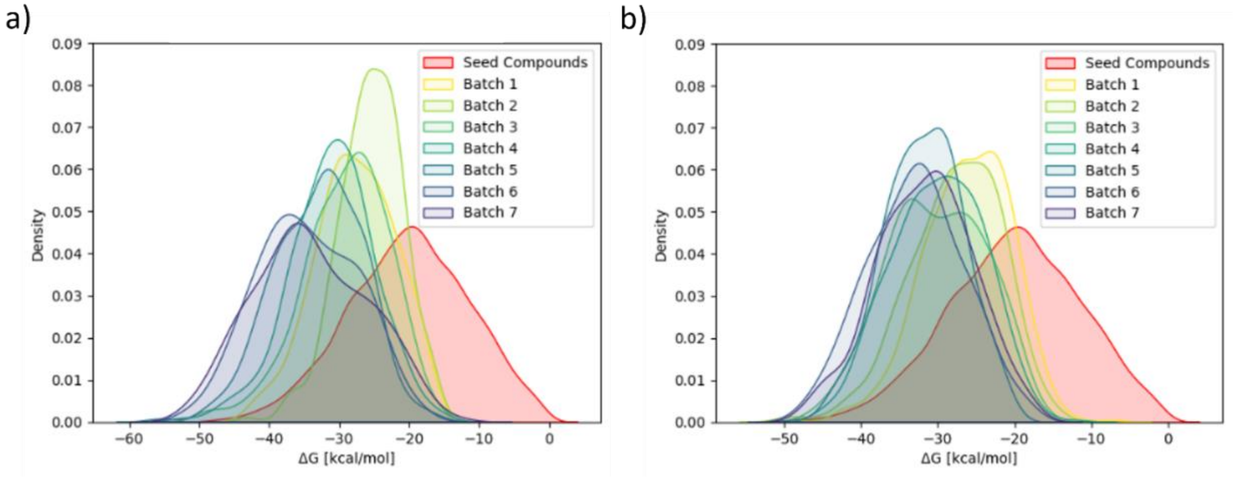

Figure S4. Distribution of calculated ΔG_{ESMACS} for each GAL iteration for (a) batch size 250 and for (b) batch size 500 for $3CL^{pro}$. The ΔG_{ESMACS} distribution of seed compounds used to train the initial surrogate model is shown in red.

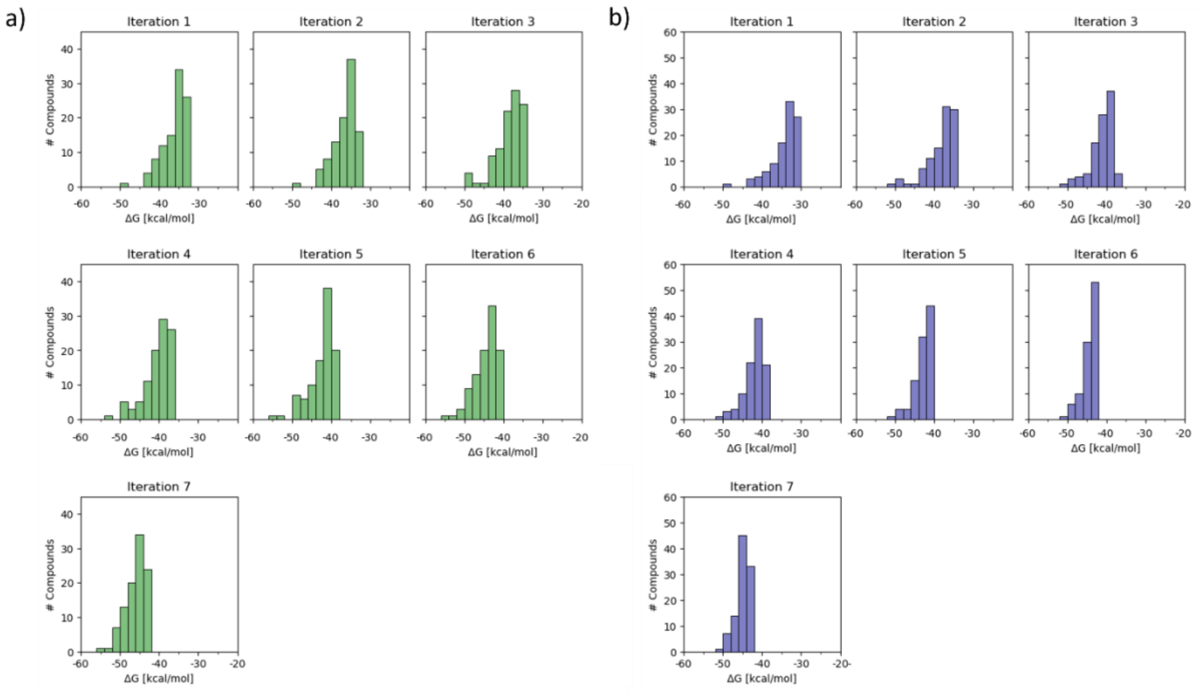

Figure S5. Distribution of calculated ΔG_{ESMACS} for each GAL iteration for $3CL^{pro}$ and for (a) batch size 250 in green and for (b) batch size 500 in blue, where only 100 compounds with the lowest ΔG_{ESMACS} were considered and taken from the accumulated pool of compounds after each iteration.

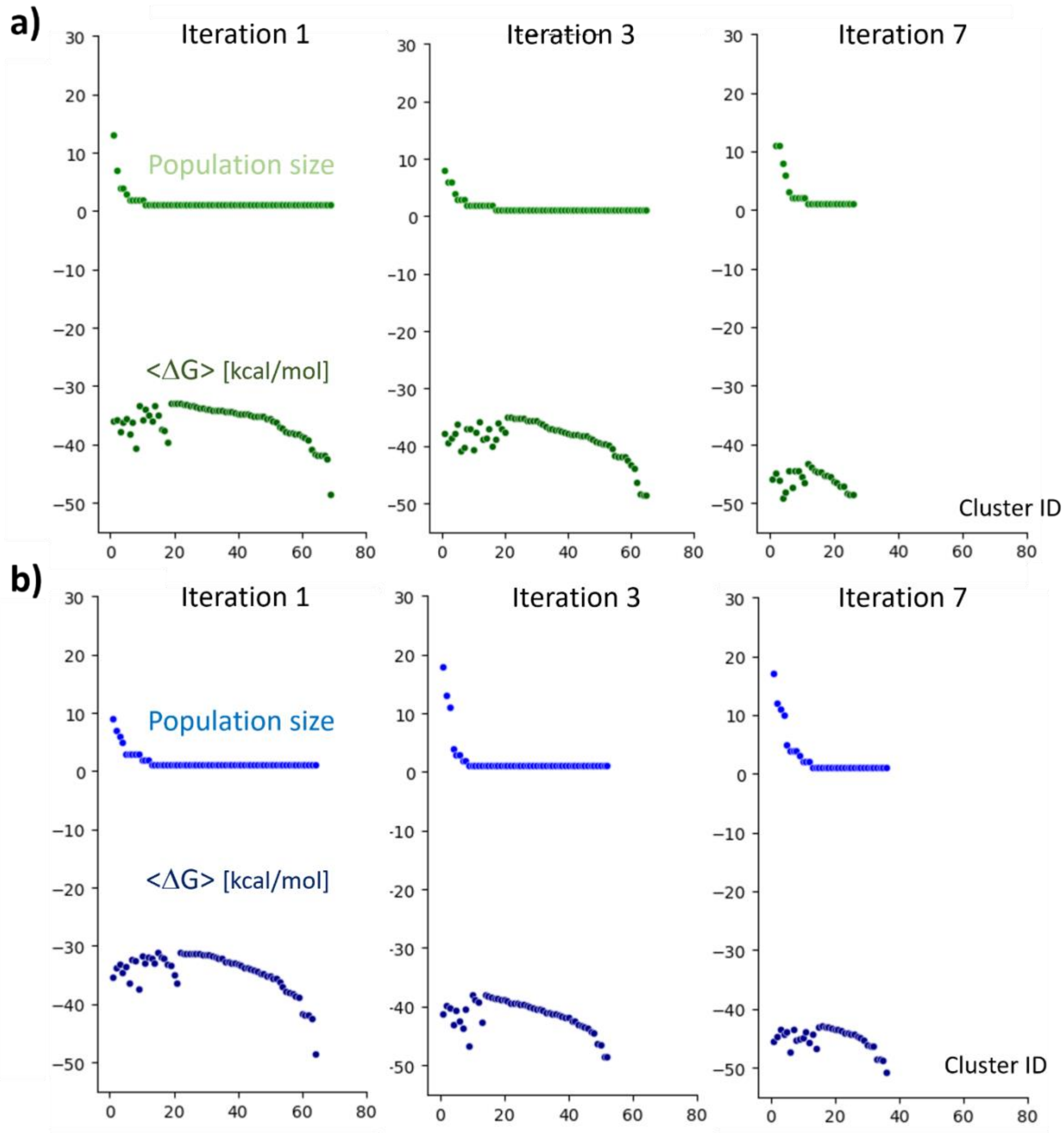

Figure S6. Average ΔG_{ESMACS} and number of molecules in each structural compound cluster for selected GAL iteration steps for (a) batch sizes 250 and (b) 500, in green and blue, respectively, for 3CL^{pro}. Only the 100 compounds with lowest ΔG_{ESMACS} were considered and taken from the accumulated pool of compounds after each iteration. Individual clusters were ordered in descending order according to their population size.

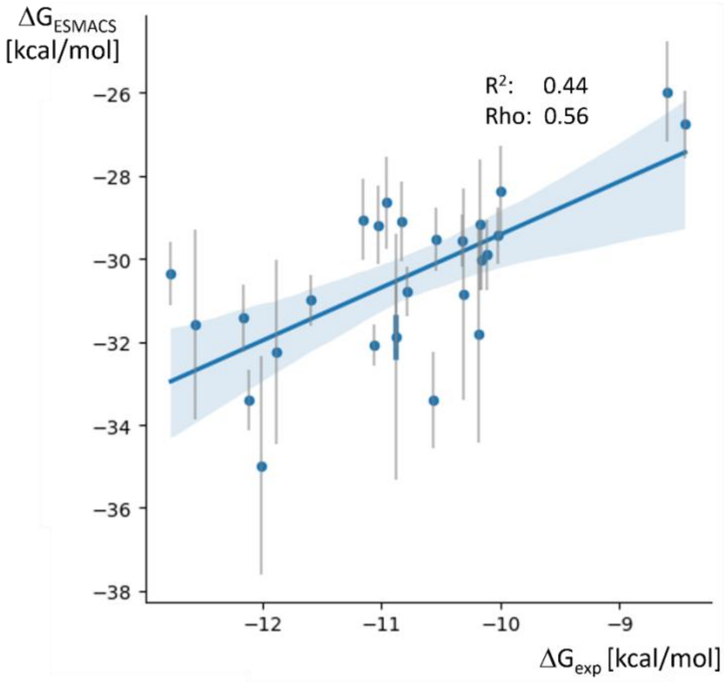

Figure S7. Comparison of binding free energies derived with ESMACS with measured values for 27 ligands to Tankyrase-2. Pearson R^2 coefficient and Spearman's rank coefficient rho are also given.

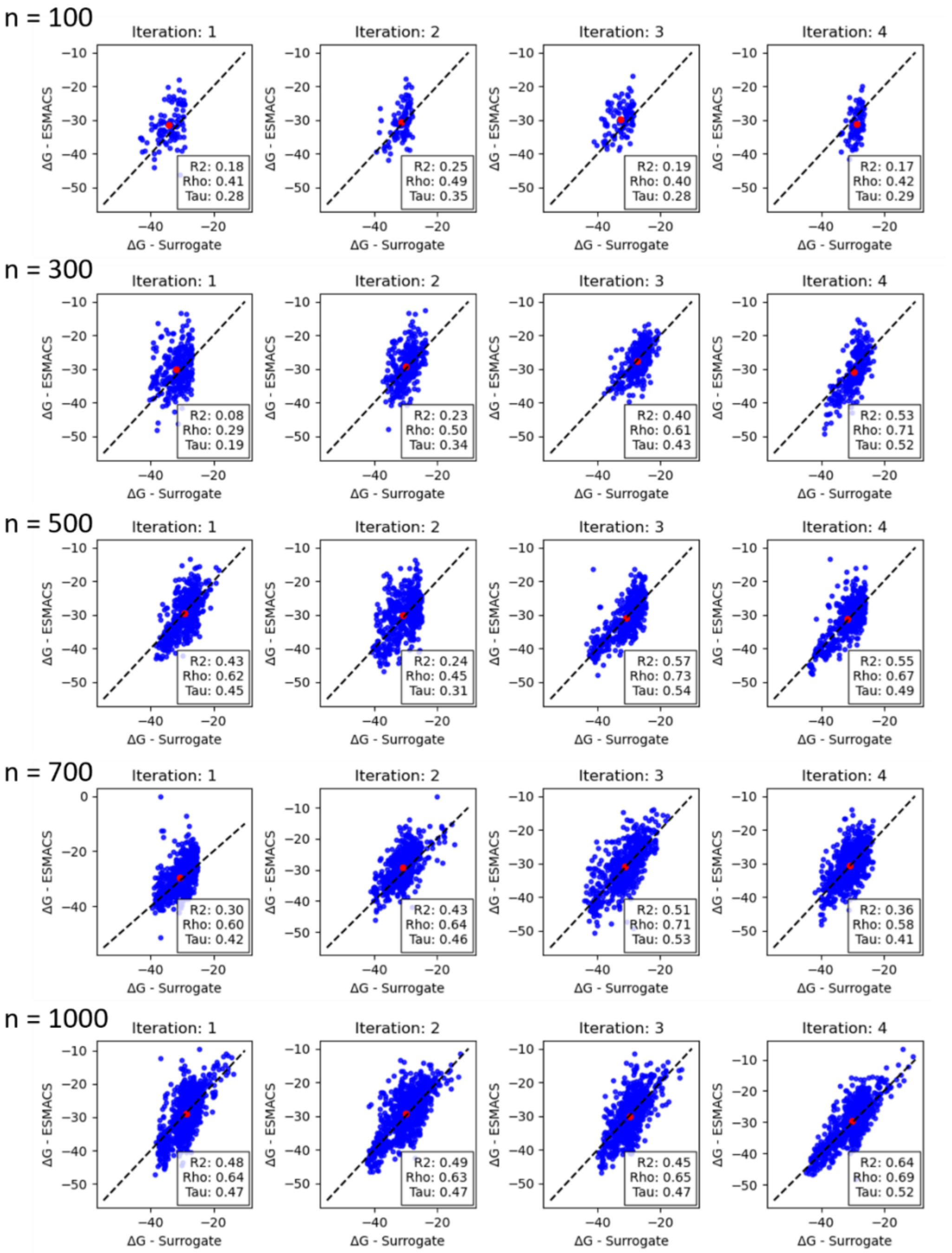

Figure S8. Comparison of surrogate model predictions of ΔG with calculated ESMACS values for training batch sizes between 100 and 1000 molecules for each GAL iteration step for Tankyrase-2. R^2-coefficient as well as Spearman and Kendall rank correlation coefficients rho and tau are given in the insets of each plot. The average ΔG of all surrogate model predictions and ESMACS calculations within an iteration is shown as a red circle. All energies are given in units of kcal/mol.

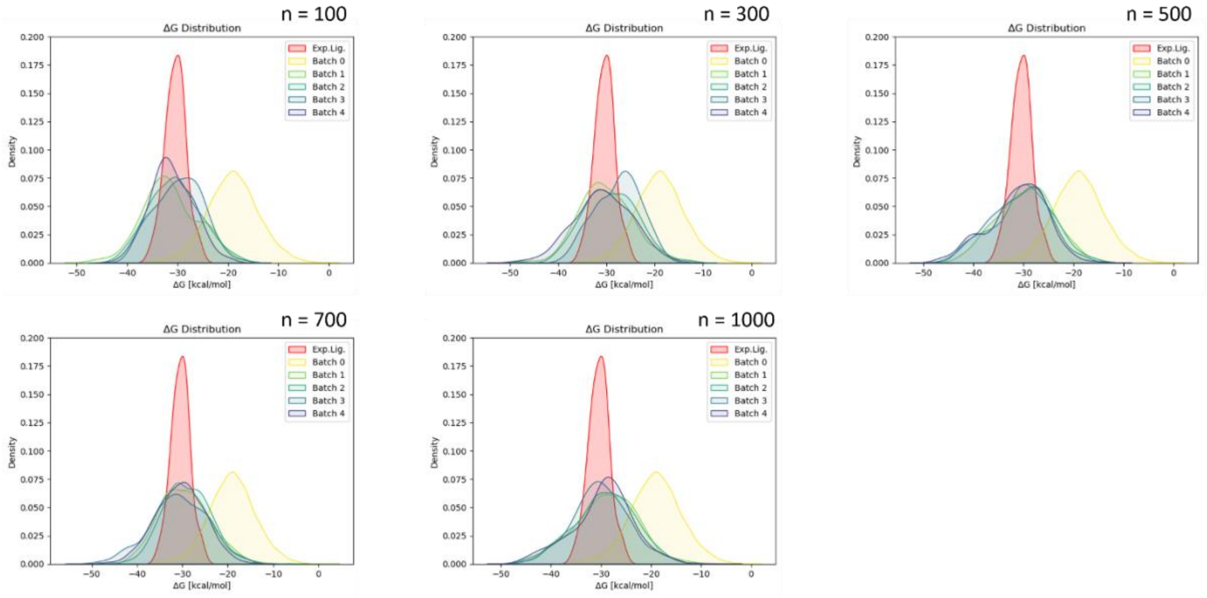

Figure S9. Distribution of calculated ΔG_{ESMACS} for each GAL iteration using different batch sizes for TNKS2. The ΔG_{ESMACS} distribution of 10k seed compounds used to train the initial surrogate model is shown in yellow as batch 0. The ΔG distribution of 27 measured compounds is shown for comparison in red.

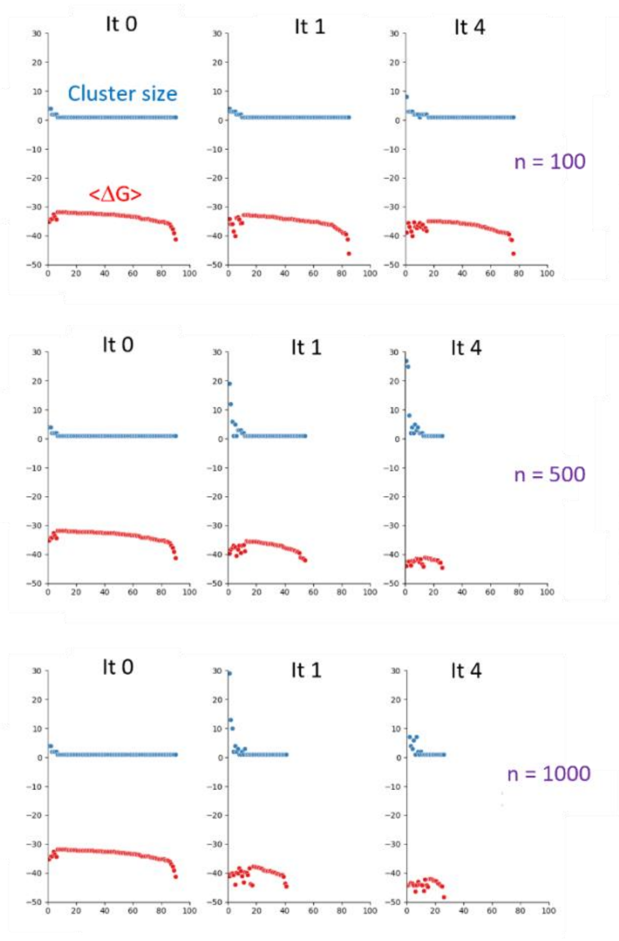

Figure S10. Average ΔG_{ESMACS} and number of molecules in each structural compound cluster for selected GAL iteration steps for different batch sizes for TNKS2. Only the 100 compounds with lowest ΔG_{ESMACS} were considered and taken from the accumulated pool of compounds after each iteration. Individual clusters were ordered in descending order according to their population size.

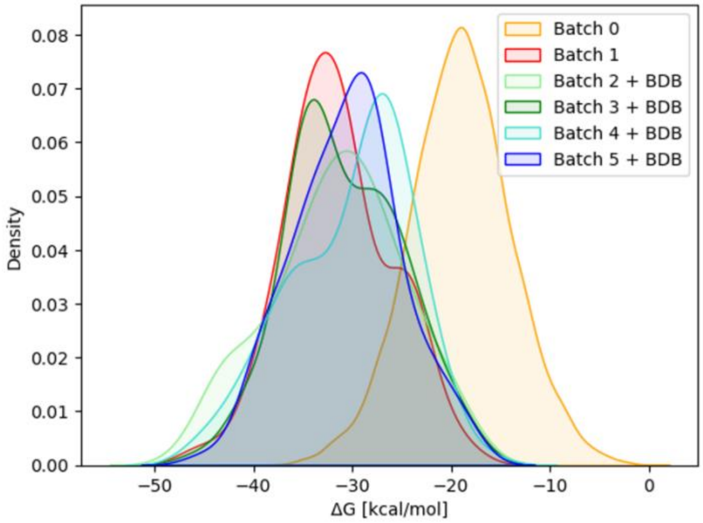

Figure S11. Distribution of calculated ΔG_{ESMACS} for each GAL iteration for a batch size of 100, for TNKS2. From iteration 2 onwards, generated structures were enriched with new ligands taken from BindingDB to increase structural diversity. Iterations 1 and 2, shown in orange and red, respectively, correspond to the same GAL steps as shown in Fig. 10. No improvements were achieved with structure infusions from BindingDB.

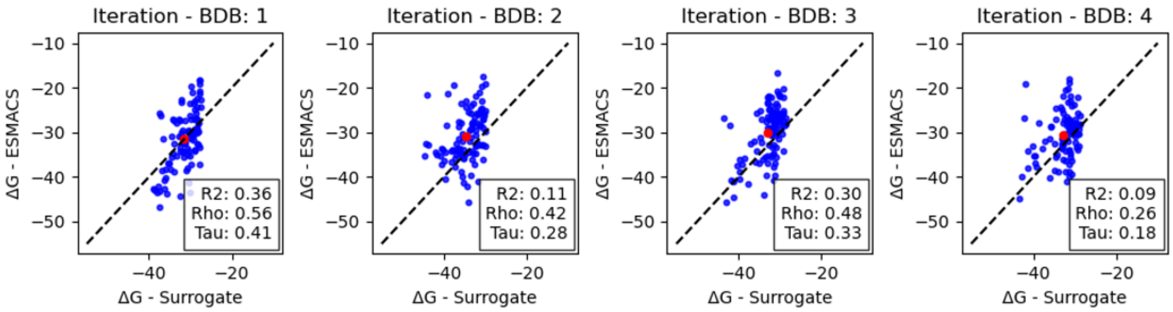

Figure S12. Comparison of surrogate model predictions of ΔG with calculated ESMACS values for training batch sizes of 100 molecules for each GAL iteration step for $3CL^{pro}$. R^2-coefficient as well as Spearman and Kendall rank correlation coefficients rho and tau are given in the insets of each plot. The average ΔG of all surrogate model predictions and ESMACS calculations within an iteration is shown as a red circle. All energies are given in units of kcal/mol. The quality of the surrogate model is similar to the results shown in Fig. 9 for n = 100.

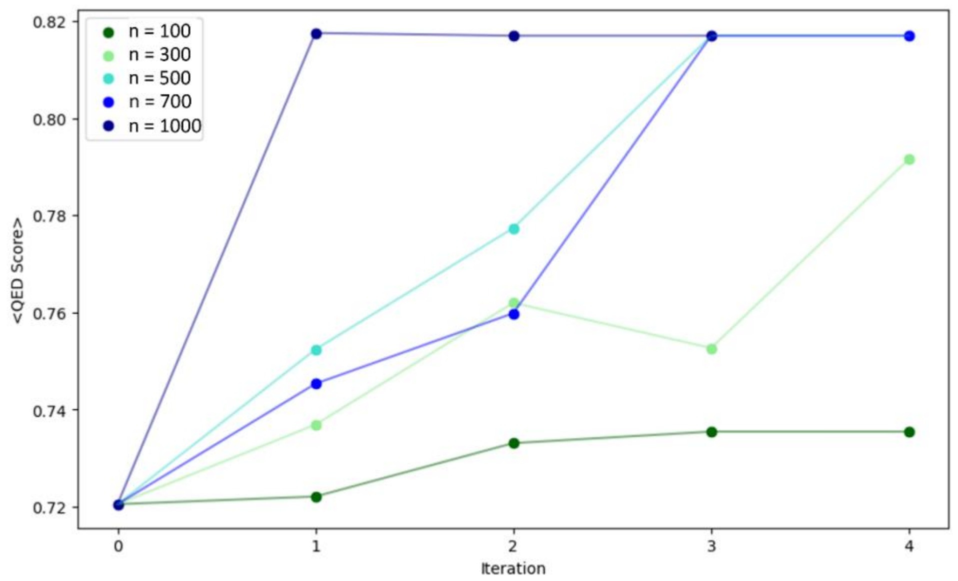

Figure S13. Average QED score, i.e. drug-likeness, for different learning batch sizes used for TNKS2. Only the 100 compounds with lowest ΔG_{ESMACS} were considered and taken from the accumulated pool of compounds after each iteration.

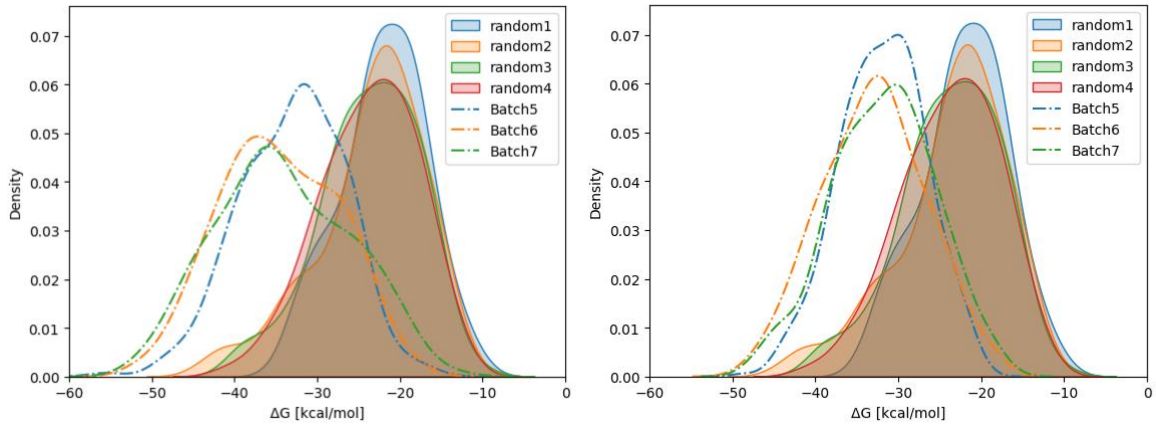

Figure S14. Comparing cluster-greedy acquisition (dash-dotted lines) with random acquisition (full lines and filled shapes) for 3CLPro. Random compound selection is much less efficient in finding high-scoring (low ΔG_{ESMACS}) compounds. Comparison with batch size 250 (left) and batch size 500 (right). The random acquisition results have been computed with a batch size of 100.

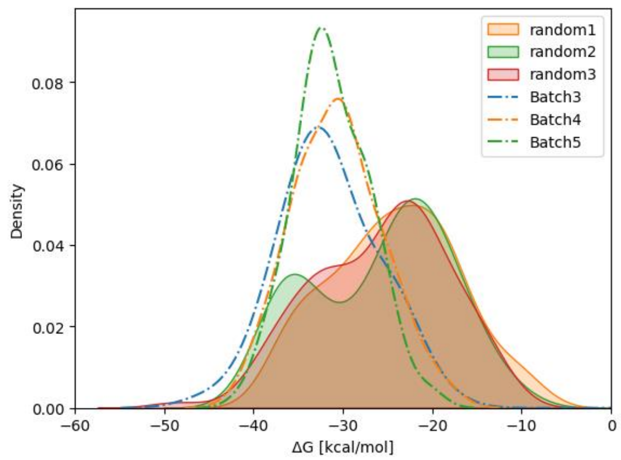

Figure S15. Comparing cluster-greedy acquisition (dash-dotted lines) with random acquisition (full lines and filled shapes) for TNKS2. Random compound selection is less efficient in finding high-scoring (low ΔG_{ESMACS}) compounds. Comparison with batch size 100. The random acquisition results have been computed with a batch size of 100.
